# Mother optimization algorithm: a new human-based metaheuristic approach for solving engineering optimization

**DOI:** 10.1038/s41598-023-37537-8

**Published:** 2023-06-26

**Authors:** Ivana Matoušová, Pavel Trojovský, Mohammad Dehghani, Eva Trojovská, Juraj Kostra

**Affiliations:** 1 Department of Mathematics, Faculty of Science, University of Hradec Králové, Rokitanského 62, 500 03 Hradec Králové, Czech Republic; 2 Faculty of Chemical Technology, Institute of Applied Physics and Mathematics, University of Pardubice, 532 10 Pardubice, Czech Republic

**Keywords:** Engineering, Mathematics and computing

## Abstract

This article’s innovation and novelty are introducing a new metaheuristic method called mother optimization algorithm (MOA) that mimics the human interaction between a mother and her children. The real inspiration of MOA is to simulate the mother’s care of children in three phases education, advice, and upbringing. The mathematical model of MOA used in the search process and exploration is presented. The performance of MOA is assessed on a set of 52 benchmark functions, including unimodal and high-dimensional multimodal functions, fixed-dimensional multimodal functions, and the CEC 2017 test suite. The findings of optimizing unimodal functions indicate MOA’s high ability in local search and exploitation. The findings of optimization of high-dimensional multimodal functions indicate the high ability of MOA in global search and exploration. The findings of optimization of fixed-dimension multi-model functions and the CEC 2017 test suite show that MOA with a high ability to balance exploration and exploitation effectively supports the search process and can generate appropriate solutions for optimization problems. The outcomes quality obtained from MOA has been compared with the performance of 12 often-used metaheuristic algorithms. Upon analysis and comparison of the simulation results, it was found that the proposed MOA outperforms competing algorithms with superior and significantly more competitive performance. Precisely, the proposed MOA delivers better results in most objective functions. Furthermore, the application of MOA on four engineering design problems demonstrates the efficacy of the proposed approach in solving real-world optimization problems. The findings of the statistical analysis from the Wilcoxon signed-rank test show that MOA has a significant statistical superiority compared to the twelve well-known metaheuristic algorithms in managing the optimization problems studied in this paper.

## Introduction

In the realm of science, problems that have multiple feasible solutions are referred to as optimization problems. Therefore, finding the best feasible solution among all the available solutions for a problem is called the optimization process^1^. Mathematically, any optimization problem can be represented using three key components: decision variables, constraints, and objective functions^2^. Problem-solving methods for addressing optimization problems can be categorized into two main groups: deterministic and stochastic techniques^3^. Deterministic methods effectively solve simple, linear, convex, continuous, differentiable, and low-dimensional optimization problems. However, they can become inefficient when dealing with complex optimization problems and may get stuck in local optima instead of finding the global optimum solution^4^. Optimization problems in science, engineering, and real-world applications often have nonlinear, nonconvex, discontinuous, nondifferentiable, and high-dimensional characteristics. The limitations and challenges of deterministic approaches have prompted researchers to develop stochastic methods for solving optimization problems. These stochastic approaches offer a more flexible and robust framework that can better handle the complexity and uncertainty of these types of issues^5^. It employs a random search in the problem-solving space and uses random operators to provide appropriate solutions for optimization problems. Metaheuristic algorithms have many advantages, including simple concepts, easy implementation, and the ability to efficiently solve nonlinear, nonconvex, discontinuous, nondifferentiable, high-dimensional, and NP-hard problems, as well as problems in nonlinear and unknown search spaces. These advantages have made metaheuristic methods popular among researchers^6^. In metaheuristic algorithms, the optimization process randomly generates a set of candidate solutions. These solutions are then improved iteratively based on specific update steps until the best solution is found. Finally, this best solution is used to solve the optimization issue^7^. One important thing to note about metaheuristic algorithms is that, unlike deterministic approaches, there is no guarantee that they will find the globally optimal solution. The reason for this is the stochastic nature of these algorithms, which rely on a random search to explore the problem space. However, even if the optimal global solution is not found, the solutions obtained from metaheuristic algorithms are usually still acceptable as quasi-optimal because they tend to be close to the optimal global solution. Metaheuristic techniques are used to solve optimization problems by searching the problem-solving space globally and locally^8^. Global search, or exploration, involves comprehensively scanning the search space to discover the main optimal area and prevent getting stuck in local optima. Local search, or exploitation, involves achieving better solutions around the obtained solutions. Metaheuristic algorithms must be able to balance exploration and exploitation during the search process, to bring usable solutions for optimization problems. This balance is the key to the success of metaheuristic algorithms in achieving suitable solutions for optimization problems^9^.

The difference in updating steps and the search process can lead to varying results when implementing metaheuristic algorithms on the same optimization problem. Hence, when comparing the performance of multiple metaheuristic algorithms on an issue, the one that performs the search process more effectively and provides a better solution will outperform others. Researchers have developed numerous metaheuristic algorithms to solve optimization problems more effectively. These methods have found applications in various fields such as dynamic scheduling^10^, construction of multi-classifier systems^11, 12^, clustering approach^13–15^, IoT-based complex problems^16, 17^, parameter estimation^18–20^, modeling of nonlinear processes^21, 22^, energy carriers and electrical engineering^23–27^, wave solutions^28–31^, and higher-order nonlinear dynamical equation^32^.

The central inquiry in investigating metaheuristic algorithms is whether the existing multitude of algorithms designed thus far is sufficient or if there is a continued need to develop newer algorithms. The No Free Lunch (NFL) theorem^33^ answers this open issue by stating that the superior performance of a particular metaheuristic algorithm in solving a specific set of optimization problems does not necessarily ensure that the same algorithm will perform similarly well in solving other optimization problems. One metaheuristic algorithm may succeed in converging to the optimal global solution for a particular optimization problem but may fail to do so for another issue. Therefore, it cannot be assumed that a given metaheuristic algorithm will successfully solve any optimization problem. The NFL states that no single metaheuristic algorithm is the best optimizer for all optimization problems. This theorem motivates researchers to develop new metaheuristic algorithms that effectively solve specific optimization problems. For instance, the authors of this paper were inspired by the NFL theorem to design a new metaheuristic algorithm that can solve optimization problems in various scientific and real-world applications.

The innovation and novelty of this paper are in introducing a new metaheuristic algorithm called mother optimization algorithm (MOA) to solve optimization problems in different sciences. This paper’s principal achievements are:

- MOA is to simulate the interactions between a mother and her children in three phases: education, advice, and upbringing.
- The MOA's performance is assessed by testing it on 52 standard benchmark functions, including unimodal, high-dimensional multimodal, fixed-dimensional multimodal, and CEC 2017 test suite.
- MOA has demonstrated significantly better performance when solving various optimization problems from the CEC 2017 test suite compared to twelve commonly used metaheuristic algorithms.
- MOA’s effectiveness in solving real-world optimization problems was tested by applying it to four engineering design problems.

The structure of the remaining sections in the paper is as follows: a literature review is presented in the “Literature review” section, followed by the introduction and modeling of the proposed MOA approach in the “Mother optimization algorithm” section. The discussion, advantages, and limitations of MOA are provided in the “Discussion” section. Simulation studies and results are summarized in the “Simulation analysis and results” section, while the efficiency of MOA in handling real-world applications is evaluated in the “MOA for real-world applications” section. Finally, conclusions are drawn, and suggestions for future work are provided in the “Conclusion and future works” section.

## Literature review

Metaheuristic algorithms are designed and developed with inspiration from various natural phenomena, the behavior of living organisms, biological sciences, physical laws, rules of games, human interactions, and other evolutionary phenomena. Based on the main design idea, metaheuristic algorithms can be classified into five groups: swarm-based, evolutionary-based, physics-based, game-based, and human-based approaches.

Swarm-based metaheuristic techniques draw inspiration from the collective behavior of social animals, plants, insects, and other organisms to develop powerful optimization methods. Particle swarm optimization (PSO)^34^, ant colony optimization (ACO)^35^, artificial bee colony (ABC)^36^, and firefly algorithm (FA)^37^ are among the most widely recognized swarm-based metaheuristic algorithms.

PSO was inspired by the swarm movement of birds and fish in search of food, while ACO was inspired by the ability of ants to identify the shortest path between the nest and food sources. ABC algorithm is inspired by the foraging behavior of honey bees in the colony. In contrast, the flashing behavior of fireflies and their optical communication have served as a basis for creating the FA algorithm. Among the natural behaviors of living organisms, searching for food, foraging, hunting strategy, and migration are intelligent processes that inspired models of many swarm-based metaheuristic algorithms such as grey wolf optimization (GWO)^38^, emperor penguin optimizer (EPO)^39^, pelican optimization algorithm (POA)^40^, rat swarm optimization (RSO)^41^, marine predators algorithm (MPA)^42^, African vultures optimization algorithm (AVOA)^43^, mutated leader algorithm (MLA)^44^, coati optimization algorithm (COA)^45^, tunicate swarm algorithm (TSA)^46^, termite life cycle optimizer (TLCO)^47^, two stage optimization (TSO)^48^, artificial hummingbird algorithm (AHA)^49^, fennec fox optimization (FFA)^50^, white shark optimizer (WSO)^51^, and reptile search algorithm (RSA)^52^.

Metaheuristic algorithms based on evolutionary principles have drawn inspiration from biological sciences, genetics, and the idea of natural selection. Genetic algorithm (GA)^53^ and differential evolution (DE)^54^ are the most famous Evolutionary-based metaheuristic methods that have been used to solve many optimization problems. GA and DE are developed based on modeling the reproduction process, Darwin’s evolutionary theory, survival of the fittest, concepts of genetics and biology, and the application of random selection, crossover, and mutation operators. Some other evolutionary-based metaheuristic algorithms are artificial immune system (AIS)^55^, biogeography-based optimizer (BBO)^56^, cultural algorithm (CA)^57^, evolution strategy (ES)^58^, and genetic programming (GP)^59^.

Metaheuristic algorithms based on physics have been designed by drawing inspiration from concepts, phenomena, laws, and forces in physics. Simulated Annealing (SA), for example, is a well-known physics-based metaheuristic algorithm that was inspired by the annealing phenomenon of metals in which the metal is melted under heat and then slowly cooled to form an ideal crystal^60^. Algorithms such as gravitational search algorithm (GSA)^61^ have been designed based on inspiration from physical forces, particularly the gravitational force. The concept of abnormal oscillations in water turbulent flow was the basis for the turbulent flow of water-based optimization (TFWO)^62^. Concepts from cosmology have inspired algorithms such as multi-verse optimizer (MVO)^63^, black hole (BH)^64^, and galaxy-based search algorithm (GbSA)^65^. Some other physics-based algorithms are magnetic optimization algorithm (MOA)^66^, artificial chemical reaction optimization algorithm (ACROA)^67^, ray optimization (RO) algorithm^68^, and small world optimization algorithm (SWOA)^69^.

Metaheuristic algorithms inspired by the rules and behaviors of players, coaches, and referees in individual and group games have been proposed as game-based metaheuristic algorithms. League championship algorithm (LCA)^70^, football game based optimizer (FGBO)^71^, and volleyball premier league (VPL)^72^ are examples of game-based metaheuristic algorithms that simulate the rules and behavior of football and volleyball league matches, respectively.

The main inspiration behind the puzzle optimization algorithm (POA)^73^ design has been the skill and accuracy required to assemble puzzle pieces. The strategy used by players to throw darts and score points has been the primary source of inspiration for designing the Darts Game Optimizer (DGO)^74^.

Inspiration from human interactions, communication, thoughts, and relationships in personal and social life has led to the development of human-based metaheuristic algorithms. One such algorithm is teaching–learning based optimization (TLBO), which simulates educational interactions between teachers and students in the classroom^75^. Teaching–learning-studying-based optimizer (TLSBO)^76^ is a method that enhances TLBO by adding a new strategy called “studying strategy”, in which each member uses the information from another randomly selected individual to improve its position. Dynamic group strategy TLBO (DGSTLBO)^77^ is an improved TLBO algorithm that enables each learner to learn from the mean of his corresponding group. Distance-fitness learning TLBO (DFL-TLBO)^78^ variant that employs a brand-new distance-fitness learning (DFL) strategy to enhance searchability. Learning cooking skills in training courses has inspired the design of the chef-based optimization algorithm (CBOA)^79^. The election based optimization algorithm (EBOA) has been inspired by the concept of elections and voting, with the aim of designing an algorithm that mimics the voting process to find optimal solutions^80^. Driving training-based optimization (DTBO)^81^, coronavirus herd immunity optimizer (CHIO)^82^, political optimizer (PO)^83^, brain storm optimization (BSO)^84^, and war strategy optimization (WSO)^85^ are among the other human-based metaheuristic algorithms that have been proposed, inspired by various aspects of human behavior and social interactions.

As far as the literature review suggests, no metaheuristic algorithm has been developed so far that models the interactions among humans in the context of mothers’ care for children. The high level of intelligence involved in a mother's care of her children presents a promising opportunity for the design of a novel metaheuristic algorithm. This paper aims to fill the research gap by proposing a novel metaheuristic algorithm that models human interactions between mothers and their children. The details of this new algorithm will be presented in the following section.

## Mother optimization algorithm

This section will introduce the mother optimization algorithm (MOA) and its mathematical model. This section aims to present MOA and its underlying mathematical framework comprehensively. By delving into the algorithm's details and mathematical representation, readers will gain insights into MOA's inner workings and principles.

### Introducing the mother optimization algorithm (MOA)

The first place of education in society is undoubtedly the family, and the mother is the essential educational element in raising children^86^. A mother passes her meaningful life experiences and skills to her children, who develop their abilities based on her advice^87^.

Among the most significant types of interactions between a mother and her children are the three processes of (i) education, (ii) advice, and (iii) upbringing. Therefore, the proposed MOA uses mathematical modeling of caring and educational behaviors.

### Mathematical model of MOA

The proposed MOA is a population-based metaheuristic algorithm that solves optimization problems through an iterative process. The algorithm’s population consists of candidate solutions represented as vectors in the problem space. The population is modeled as a matrix by Eq. (1) and initialized using Eq. (2) at the start of the optimization process. Each member of the population determines the values of decision variables based on its position in the problem search space, and the search power of the population is used to find the optimal solution.

1 $${\varvec{X}} = \left[ {\begin{array}{*{20}c} {X_{1} } \\ \vdots \\ {X_{i} } \\ \vdots \\ {X_{N} } \\ \end{array} } \right]_{N \times m} = \left[ {\begin{array}{*{20}c} {x_{1,1} } & \cdots & {x_{1,j} } & \cdots & {x_{1,m} } \\ \vdots & \ddots & \vdots & {\mathinner{\mkern2mu\raise1pt\hbox{.}\mkern2mu \raise4pt\hbox{.}\mkern2mu\raise7pt\hbox{.}\mkern1mu}} & \vdots \\ {x_{i,1} } & \cdots & {x_{i,j} } & \cdots & {x_{i,m} } \\ \vdots & {\mathinner{\mkern2mu\raise1pt\hbox{.}\mkern2mu \raise4pt\hbox{.}\mkern2mu\raise7pt\hbox{.}\mkern1mu}} & \vdots & \ddots & \vdots \\ {x_{N,1} } & \cdots & {x_{N,j} } & \cdots & {x_{N,m} } \\ \end{array} } \right]_{N \times m} ,$$

2 $${x}_{i,j}=l{b}_{j}+\mathrm{rand}\left(\mathrm{0,1}\right)\cdot \left(u{b}_{j}-l{b}_{j}\right), i=\mathrm{1,2}, \dots , N, j=\mathrm{1,2}, \dots , m,$$

where ${\varvec{X}}$ is the population matrix of the proposed MOA, $N$ is the number of population members, $m$ is the number of decision variables, ${X}_{i}=\left({x}_{i,1},\dots ,{x}_{i,j},\dots ,{x}_{i,m}\right)$ is the $i$th candidate solution, ${x}_{i,j}$ is its $j$th variable the function $\mathrm{rand}(\mathrm{0,1})$ generates a random uniform number from the interval $\left[0, 1\right].$ The $j$th decision variable's lower and upper bounds are respectively represented by $l{b}_{j}$ and $u{b}_{j}$.

Each member of the population in MOA is a potential solution to the problem being optimized, and the objective function of the problem can be computed based on the values proposed by each population member for the decision variables. In mathematical terms, the values of the objective function can be represented as a vector using Eq. (3).

3 $$F={\left[\begin{array}{c}{F}_{1}\\ \vdots \\ {F}_{i}\\ \vdots \\ {F}_{N}\end{array}\right]}_{N\times 1}={\left[\begin{array}{c}F({X}_{1})\\ \vdots \\ F({X}_{i})\\ \vdots \\ F({X}_{N})\end{array}\right]}_{N\times 1},$$

where $F$ is the vector of values of the objective function and ${F}_{i}$ is the value of the objective function for the $i$th candidate solution.

The objective function values provide a measure of the quality of the solutions generated by the population members. The best and worst population members can be identified based on the best and worst values of the objective function, respectively. As the population members’ positions are updated in each iteration, the best population member also needs to be updated accordingly. Finally, at the end of the algorithm's iterations, the best population member solves the problem.

In the design of MOA, the algorithm population is updated in three phases based on the mathematical modeling of the interaction of raising children by the mother, which is discussed below.

### Phase 1: education (exploration phase)

The first phase, called “Education,” of population update in the proposed MOA approach is inspired by children’s education. It aims to increase global search and exploration capabilities by making significant changes in the position of the population members. The mother in the MOA design is considered the best member of the population, and her behavior in training her children is modeled to simulate the education phase. In this phase, a new position for each member is created using Eq. (4). If the objective function value improves in the new position, it is accepted as the corresponding member’s position, as shown in Eq. (5).

4 $${x}_{i,j}^{P1}={x}_{i,j}+\mathrm{rand}(\mathrm{0,1}) \cdot ({M}_{j}-\mathrm{rand}(2) \cdot {x}_{i,j}),$$

5 $${X}_{i}=\left\{\begin{array}{ll}{X}_{i}^{P1}, &\quad {F}_{i}^{P1}\le {F}_{i},\\ {X}_{i}, & \quad else ,\end{array}\right.$$

where ${M}_{j}$ is its $j$th dimension of the position of the mother, ${x}_{i,j}$ is the $j$th dimension of the position of the $i$th population member ${X}_{i}$, ${X}_{i}^{P1}$ is the new position calculated for the $i$th population member based on the first phase of the MOA, ${x}_{i,j}^{P1}$ is its $j$th dimension, ${F}_{i}^{P1}$ is its objective function value, the function $\mathrm{rand}(\mathrm{0,1})$ generates a random uniform number in the interval $\left[0, 1\right]$, and $\mathrm{rand}(2)$ is the random function that uniformly generates a random number from the set $\left\{1, 2\right\}$.

### Phase 2: advice (exploration phase)

One of the primary duties of mothers in raising their children is to counsel them and not enable them to misbehave. This action of the mother in the children’s advice is employed in the design of the second phase of population update in the MOA. The advice phase leads to an increase in the MOA’s capability in global search and exploration by making significant changes in the location of the population members. In MOA design, for each member of the population, the position of other population members with a greater value of the objective function than it has is considered deviant behavior that should be avoided. The set of bad behavior ${BB}_{i}$ for each member is determined by comparing the objective function value using Eq. (6). For each ${X}_{i}$, a member is uniformly randomly selected from the constructed set of bad behaviors ${BB}_{i}$. First, a new position is created for each member using Eq. (7) to simulate keeping the child away from bad behavior. Subsequently, if it improves the objective function’s value, this new position replaces the corresponding member’s previous position, by Eq. (8).

6 $${BB}_{i}=\left\{{X}_{k}, {F}_{k}>{F}_{i} \wedge k \in \left\{\mathrm{1,2},\dots ,N\right\} \right\} , \quad \mathrm{where} \; i=\mathrm{1,2},\dots ,N,$$

7 $${x}_{i,j}^{P2}={x}_{i,j}+\mathrm{rand}(\mathrm{0,1}) \cdot ({x}_{i,j}-\mathrm{rand}(2) \cdot SB{B}_{i,j}),$$

8 $${X}_{i}=\left\{\begin{array}{ll}{X}_{i}^{P2},&\quad {F}_{i}^{P2}\le {F}_{i} ;\\ {X}_{i},&\quad else ,\end{array}\right.$$

where ${BB}_{i}$ is the set of bad behavior for the $i$th population member, $SB{B}_{i}$ is the selected bad behavior for the $i$th population member, $SB{B}_{i,j}$ is its $j$th dimension, ${X}_{i}^{P2}$ is the new position calculated for the $i$th population member based on second phase of the proposed MOA, ${x}_{i,j}^{P2}$ is its $j$th dimension, ${F}_{i}^{P2}$ is its objective function value, the function $\mathrm{rand}(\mathrm{0,1})$ generates a random uniform number in the interval $\left[0, 1\right]$, and $\mathrm{rand}(2)$ is the random function that uniformly generates a random number from the set $\left\{1, 2\right\}$.

### Phase 3: upbringing (exploitation phase)

Mothers use various forms of encouraging children to improve their skills in the education process. The upbringing leads to an increase in the ability of local search and exploitation in the MOA phase by making small changes in the position of the population members. To simulate the upbringing phase, first, a new position is created for each member of the population based on the modeling of children's personality development using Eq. (9). If the objective function value improves in the new position, the corresponding member's previous position is replaced with the new one, as specified in Eq. (10).

9 $${x}_{i,j}^{P3}={x}_{i,j}+\left(1-2\cdot \mathrm{rand}(\mathrm{0,1})\right)\cdot \frac{u{b}_{j}-l{b}_{j}}{t} ,$$

10 $${X}_{i}=\left\{\begin{array}{ll}{X}_{i}^{P3}, &\quad {F}_{i}^{P3}\le {F}_{i} ;\\ {X}_{i}, &\quad else,\end{array}\right.$$

where ${X}_{i}^{P3}$ is the new position calculated for the $i$th population member based on third phase of the proposed MOA, ${x}_{i,j}^{P3}$ is its $j$th dimension, ${F}_{i}^{P3}$ is its objective function value, the function $\mathrm{rand}(\mathrm{0,1})$ generates a random number in the interval $\left[0, 1\right]$, and $t$ is the actual value of the iteration counter.

### Description of the repetition process, pseudo-code, and flowchart of MOA

**Figure 1:**
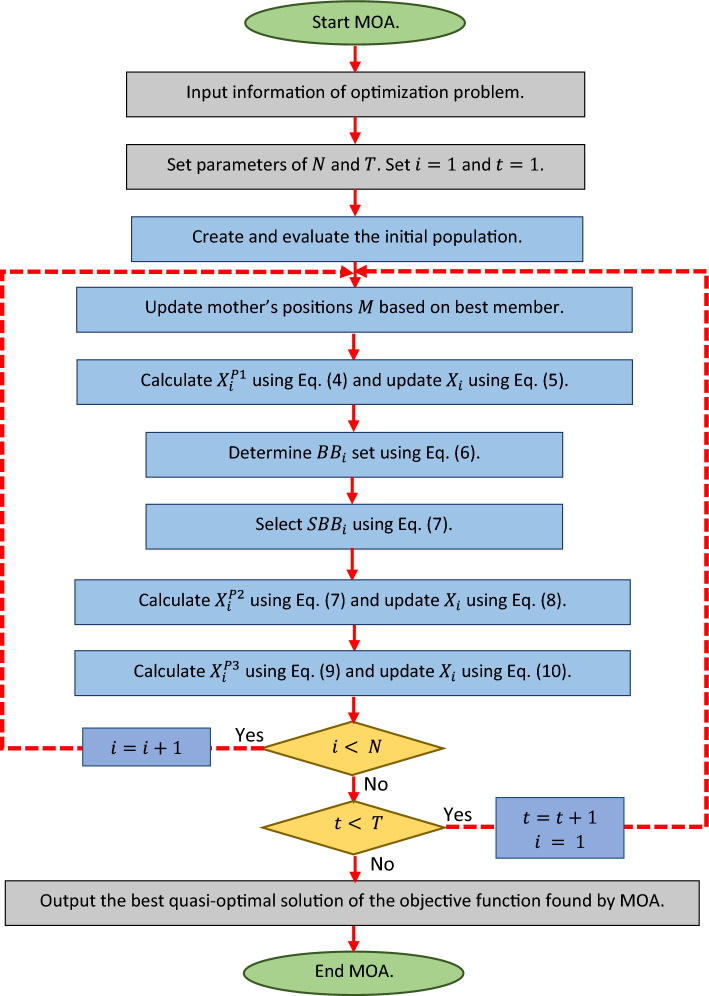
Flowchart of MOA.


After completing each iteration of the MOA algorithm, all population members are updated based on Phases 1 to 3—this process of updating the population according to Eqs. (4) to (10) continues until the final iteration. Throughout the algorithm, the best candidate solution is continuously updated and saved. Once the full implementation of the algorithm is completed, MOA presents the best candidate solution as the solution to the problem. The steps of the proposed MOA are depicted in a flowchart in Fig. 1 and pseudocode in Algorithm 1.

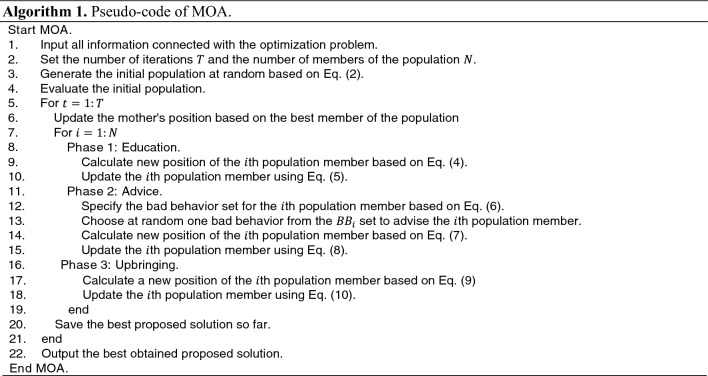


### Computational complexity of MOA

In this subsection, the MOA computational complexity analysis is discussed. MOA initialization for an optimization problem has a complexity equal to $O(Nm),$ where $N$ is the number of population members and $m$ is the number of decision variables of the problem. In each iteration, MOA population members are updated in three phases. The MOA update process has a complexity equal to $O(3NmT)$, where $T$ is the maximal number of iterations of the algorithm. Therefore, the total computational complexity of MOA is equal to $O (Nm (1+3T))$.

## Simulation analysis and results

In this section, the proposed MOA’s performance in solving optimization problems is evaluated by testing its efficiency on fifty-two standard benchmark functions, including unimodal (F1 to F7), high-dimensional multimodal (F8 to F13), and fixed-dimensional multimodal (F14 to F23) types^88^, as well as the CEC 2017 test suite (C17–F1, and C17–F3 to C17–F30)^89^. The quality of the results obtained from MOA is compared with twelve well-known metaheuristic algorithms, including GA, PSO, GSA, GWO, MVO, WOA, TSA, MPA, AVOA, WSO, and RSA. The control parameters are adjusted as specified in Table 1. To optimize functions F1 to F23, MOA and each competitor algorithm are used in twenty independent runs with 50,000 function evaluations (i.e., $\mathrm{FEs}=50,000$). For solving the CEC 2017 test set, the proposed MOA and the competitor algorithms are employed in fifty-one independent runs, each containing 1$\mathrm{10,000}\cdot m$ function evaluations (i.e., $\mathrm{FEs}=10000\cdot m$), where $m$ is the number of problem variables set to 10. The population size of MOA is considered equal to 30 members. Six statistical indicators, including mean, best, worst, standard deviation, median, and rank, are used to report the optimization results. The mean index is used as a ranking criterion for metaheuristic algorithms in optimizing each benchmark function. Experiments have been implemented on the software MATLAB R2022a using 64-bit Core i7 processor with 3.20 GHz and 16 GB main memory.

**Table 1:** Assigned values to the control parameters of competitor algorithms.

Algorithm	Parameter	Value
GA	Type	Real coded
	Selection	Roulette wheel (Proportionate)
	Crossover	Whole arithmetic ($\mathrm{Probability} = 0.8$, $\alpha \in \left[-0.5, 1.5\right]$)
	Mutation	Gaussian ($\mathrm{Probability} = 0.05$)
PSO	Topology	Fully connected
	Cognitive and social constant	$\left({C}_{1}, {C}_{2}\right)=\left(2, 2\right)$
	Inertia weight	Linear reduction from 0.9 to 0.1
	Velocity limit	10% of the dimension range
PSO	$\mathrm{Alpha}$, ${G}_{0},{R}_{\mathrm{norm}},{R}_{\mathrm{power}}$	20, 100, 2, 1
TLBO	${T}_{F}$: the teaching factor	${T}_{F} = \mathrm{round} \left[(1+rand)\right]$
	Random number *rand*	*rand* is a random number from the interval $\left[0, 1\right]$
GWO	Convergence parameter (*a*)	$a$: Linear reduction from 2 to 0
MVO	Wormhole existence probability ($\mathrm{WEP}$)	$\mathrm{Min}(WEP) = 0.2$ and $\mathrm{Max}(WEP)=1$
	Exploitation accuracy over the iterations ($p$)	$p = 6$
WOA	Convergence parameter $a$	$a$: Linear reduction from 2 to 0
	Parameters $r$ and $l$	$r$ is a random vector in $\left[0, 1\right]$
		$l$ is a random number in $\left[-1, 1\right]$
TSA	${P}_{min}$ and ${P}_{max}$	1, 4
	${c}_{1},{c}_{2},{c}_{3}$	Random numbers lie in the range $\left[0, 1\right].$
MPA	Constant number	$P=0.5$
	Random vector	*R* is a vector of uniform random numbers from $\left[0, 1\right]$
	Fish aggregating devices ($\mathrm{FADs}$)	$\mathrm{FADs}=0.2$
	Binary vector	$U= 0$ or 1
RSA	Sensitive parameter $\alpha$	$\alpha =0.1$
	Sensitive parameter $\beta$	$\beta =0.01$
	Evolutionary Sense ($\mathrm{ES}$)	$\mathrm{ES}$ are randomly decreasing values between 2 and − $2$
AVOA	${L}_{1}$, ${L}_{2}$	$\left({L}_{1},{L}_{2}\right)=\left(\mathrm{0.8,0.2}\right),$
	$w$	$w=2.5$
	${P}_{1}$, ${P}_{2}$,${P}_{3}$	$\left({P}_{1},{P}_{2},{P}_{3}\right)=\left(0.6, 0.4, 0.6\right)$
WSO	${F}_{min}$ and ${F}_{max}$	$\left({F}_{min},{F}_{max}\right)=\left(0.07, 0.75\right)$
	$\tau ,{a}_{0},{a}_{1},{a}_{2}$	$\left(\tau ,{a}_{0},{a}_{1},{a}_{2}\right)=\left(4.125, 6.25, 100, 0.0005\right)$

### Evaluation of unimodal benchmark functions

Table 2 presents the results of MOA and twelve competitor algorithms on seven unimodal functions F1 to F7, which are selected to evaluate the ability of metaheuristic algorithms in local search and exploitation. This evaluation aims to determine the algorithm’s ability to find the global optimum. The results show that MOA has achieved convergence to the global optimum for functions F1 to F6 with high exploitation ability. Additionally, MOA has performed the best among the competitor algorithms in solving F7. The analysis of the optimization results indicates that MOA has demonstrated superior performance in solving unimodal functions F1 to F7 due to its high ability in exploitation.

**Table 2:** Evaluation results of unimodal functions.

$F$		MOA	WSO	AVOA	RSA	MPA	TSA	WOA	MVO	GWO	TLBO	GSA	PSO	GA
F1	Mean	0	65.84207	0	0	1.92E−49	4.65E−47	1.40E−151	0.149486	1.77E−59	2.52E−74	1.33E−16	0.100856	30.4715
	Best	0	5.289861	0	0	3.80E−52	1.44E−50	9.30E−171	0.105404	1.49E−61	5.86E−77	5.35E−17	0.000486	17.90903
	Worst	0	238.6714	0	0	1.66E−48	3.30E−46	2.70E−150	0.201096	7.71E−59	2.59E−73	3.73E−16	1.396346	56.87106
	Std	0	58.09538	0	0	4.33E−49	1.10E−46	6.60E−151	0.030559	2.35E−59	6.78E−74	7.88E−17	0.342137	11.51854
	Median	0	45.37455	0	0	4.16E−50	4.27E−48	2.20E−159	0.150377	1.07E−59	1.69E−75	1.13E−16	0.00971	28.17077
	Rank	1	11	1	1	5	6	2	9	4	3	7	8	10
F2	Mean	0	2.1377	1.10E−276	0	6.96E−28	2.11E−28	2.50E−105	0.258914	1.35E−34	6.76E−39	5.48E−08	0.89461	2.785606
	Best	0	0.661815	1.30E−306	0	1.84E−29	2.02E−30	7.90E−118	0.159915	4.87E−36	8.81E−40	3.48E−08	0.045236	1.743611
	Worst	0	7.438052	2.20E−275	0	4.70E−27	1.82E−27	2.70E−104	0.364146	7.90E−34	2.44E−38	1.23E−07	2.490822	3.80275
	Std	0	1.953299	0	0	1.20E−27	5.83E−28	7.60E−105	0.069347	2.16E−34	6.14E−39	2.06E−08	0.795644	0.599756
	Median	0	1.528931	6.50E−290	0	3.51E−28	1.97E−29	3.40E−108	0.26808	6.50E−35	4.97E−39	5.12E−08	0.58358	2.738814
	Rank	1	11	2	1	7	6	3	9	5	4	8	10	12
F3	Mean	0	1784.524	0	0	2.51E−12	1.18E−10	19,939.26	15.95736	2.17E−14	3.84E−24	475.0243	387.7434	2166.814
	Best	0	1039.407	0	0	6.18E−19	1.37E−21	2062.816	5.9683	2.35E−19	2.20E−29	245.7179	21.74649	1422.763
	Worst	0	3539.57	0	0	1.43E−11	1.95E−09	34,653.75	48.89083	4.04E−13	3.60E−23	1185.13	1024.368	3455.476
	Std	0	691.1359	0	0	4.83E−12	4.80E−10	9420.548	11.85101	9.93E−14	1.19E−23	242.5098	317.5327	704.235
	Median	0	1556.732	0	0	1.83E−13	1.07E−13	20,303.94	11.86739	4.66E−16	4.04E−26	399.9344	292.7514	2098.599
	Rank	1	9	1	1	4	5	11	6	3	2	8	7	10
F4	Mean	0	17.2787	3.20E−265	0	2.98E−19	0.004418	51.76951	0.546571	1.23E−14	1.83E−30	1.234645	6.273603	2.826566
	Best	0	11.90291	0	0	3.01E−20	9.65E−05	0.903667	0.26566	6.55E−16	5.81E−32	9.89E−09	2.287977	2.214252
	Worst	0	23.8119	4.50E−264	0	9.60E−19	0.035792	91.61802	0.962084	5.73E−14	8.11E−30	4.922767	13.34688	3.988745
	Std	0	3.178756	0	0	2.52E−19	0.008746	32.60275	0.211601	1.61E−14	2.64E−30	1.527107	2.754864	0.514049
	Median	0	17.75492	2.00E−282	0	2.58E−19	0.001468	55.36903	0.530514	6.34E−15	6.52E−31	0.906041	5.876589	2.780694
	Rank	1	11	2	1	4	6	12	7	5	3	8	10	9
F5	Mean	0	10,788.6	1.43E−05	12.98563	23.30066	28.44887	27.28239	96.12534	26.55501	26.76115	44.00585	4607.322	594.79
	Best	0	1345.963	1.39E−06	8.70E−29	22.78581	25.64537	26.69534	27.6041	25.54099	25.5631	25.85872	26.25471	228.5792
	Worst	0	92,623.17	5.90E−05	28.96122	24.02522	28.86278	28.70663	377.5262	27.12889	28.72392	167.0769	89,987.2	2254.801
	Std	0	22,093.25	1.59E−05	16.23233	0.427845	0.867651	0.636008	111.7016	0.579436	1.030818	48.79555	22,146.34	467.867
	Median	0	5604.085	9.38E−06	1.22E−28	23.27164	28.79376	27.05974	29.98803	26.20545	26.30152	26.32007	86.01194	475.0975
	Rank	1	13	2	3	4	8	7	10	5	6	9	12	11
F6	Mean	0	100.8059	4.97E−08	6.451426	1.80E−09	3.678225	0.081492	0.150852	0.660188	1.260143	1.05E−16	0.063382	34.11331
	Best	0	16.93604	7.10E−09	3.659595	8.07E−10	2.55026	0.01051	0.079154	0.246482	0.232888	5.52E−17	1.90E−06	15.59683
	Worst	0	382.1118	1.36E−07	7.242753	4.80E−09	4.782888	0.326421	0.24986	1.251026	2.162628	1.81E−16	0.541189	62.70425
	Std	0	105.1108	3.62E−08	1.13166	1.03E−09	0.763317	0.111874	0.052161	0.337545	0.547394	4.08E−17	0.163552	14.91716
	Median	0	69.50695	4.61E−08	6.878069	1.60E−09	3.792199	0.031576	0.159996	0.726589	1.216208	9.47E−17	0.002055	31.6505
	Rank	1	13	4	11	3	10	6	7	8	9	2	5	12
F7	Mean	2.54E−05	9.00E−05	6.25E−05	3.01E−05	0.000546	0.004338	0.001277	0.011603	0.00083	0.001528	0.052756	0.183957	0.010578
	Best	2.35E−06	1.06E−05	8.71E−07	2.47E−06	0.000111	0.001492	2.02E−05	0.003967	0.000182	9.00E−05	0.01411	0.068948	0.003029
	Worst	6.89E−05	0.000339	0.000261	0.000133	0.000898	0.009963	0.005394	0.022546	0.001955	0.002944	0.095479	0.41094	0.021917
	Std	2.18E−05	9.85E−05	8.07E−05	3.80E−05	0.000236	0.002577	0.001591	0.005542	0.000514	0.000968	0.027476	0.086987	0.005305
	Median	1.83E−05	6.37E−05	4.01E−05	1.54E−05	0.000533	0.003717	0.000817	0.011304	0.000844	0.001505	0.05178	0.177553	0.010168
	Rank	1	4	3	2	5	9	7	11	6	8	12	13	10
Sum rank		7	72	15	20	32	50	48	59	36	35	54	65	74
Mean rank		1	10.28571	2.142857	2.857143	4.571429	7.142857	6.857143	8.428571	5.142857	5	7.714286	9.285714	10.57143
Total ranking		1	12	2	3	4	8	7	10	6	5	9	11	13

### Evaluation of high dimensional multimodal benchmark functions

Table 3 reports the optimization results of six high-dimensional multimodal functions (F8 to F13) using MOA and other competitor algorithms. The aim of selecting these functions was to evaluate the ability of metaheuristic algorithms in global search and exploration. The results show that MOA has outperformed the other algorithms and has been able to provide the global optimal for F9 and F11 functions. Additionally, MOA is the best optimizer for benchmark functions F8, F10, F12, and F13. It is observed that the proposed MOA approach, which has high power in exploration, has provided better results and superior performance in solving high-dimensional multimodal functions compared to the competitor algorithms.

**Table 3:** Evaluation results of high-dimensional multimodal functions.

$F$		MOA	WSO	AVOA	RSA	MPA	TSA	WOA	MVO	GWO	TLBO	GSA	PSO	GA
F8	Mean	− 12,498.6	− 7056.73	− 12,470.7	− 5443.28	− 9690.26	− 6145.54	− 11,066.5	− 7837.61	− 6086.06	− 5605.29	− 2790.97	− 6553.37	− 8425.58
	Best	− 12,622.8	− 9003.98	− 12,569.5	− 5663.03	− 10,477.6	− 7324.31	− 12,569.5	− 9191.67	− 6868.8	− 7033.72	− 3983.07	− 8247.98	− 9684.08
	Worst	− 11,936.3	− 6088.83	− 11,897.5	− 4917.06	− 9094.02	− 4377.98	− 7744.88	− 6885.32	− 5055.53	− 4558.05	− 2158.1	− 4996.61	− 7034.54
	Std	209.8199	808.5881	215.5843	248.2681	407.5597	803.5663	1910.15	802.0021	530.3933	670.6257	545.6289	823.9522	705.9341
	Median	− 12,577.8	− 6978.37	− 12,569.4	− 5497.94	− 9722.23	− 6104.11	− 12,041.4	− 7715.63	− 6079.35	− 5620.58	− 2702.87	− 6698.93	− 8403.34
	Rank	1	7	2	12	4	9	3	6	10	11	13	8	5
F9	Mean	0	24.60552	0	0	0	172.951	0	97.73189	1.70E−14	0	28.47705	67.64668	54.62655
	Best	0	14.60502	0	0	0	89.6551	0	52.73406	0	0	13.9155	39.75856	23.20916
	Worst	0	45.90466	0	0	0	287.8962	0	149.1313	1.14E−13	0	48.7042	114.4475	76.82396
	Std	0	9.487691	0	0	0	56.15377	0	27.73931	3.57E−14	0	10.09094	20.74215	15.20074
	Median	0	22.66603	0	0	0	166.5089	0	96.98589	0	0	26.34004	65.0035	52.56182
	Rank	1	3	1	1	1	8	1	7	2	1	4	6	5
F10	Mean	8.88E−16	5.286092	8.88E−16	8.88E−16	4.26E−15	1.24125	4.08E−15	0.577321	1.67E−14	4.44E−15	8.20E−09	2.724506	3.571525
	Best	8.88E−16	3.379557	8.88E−16	8.88E−16	8.88E−16	7.99E−15	8.88E−16	0.1005	7.99E−15	4.44E−15	4.66E−09	1.691756	2.87908
	Worst	8.88E−16	8.190507	8.88E−16	8.88E−16	4.44E−15	3.37008	7.99E−15	2.512673	2.22E−14	4.44E−15	1.44E−08	5.052015	4.637325
	Std	0	1.344712	0	0	8.75E−16	1.727866	2.51E−15	0.745512	3.91E−15	8.92E−31	2.57E−09	0.944349	0.436664
	Median	8.88E−16	5.174299	8.88E−16	8.88E−16	4.44E−15	2.22E−14	4.44E−15	0.194121	1.51E−14	4.44E−15	7.72E−09	2.731187	3.625951
	Rank	1	11	1	1	3	8	2	7	5	4	6	9	10
F11	Mean	0	1.714441	0	0	0	0.008834	0	0.399276	0.001338	0	7.200806	0.185081	1.471998
	Best	0	1.102774	0	0	0	0	0	0.253894	0	0	2.992647	0.002365	1.286807
	Worst	0	3.281444	0	0	0	0.020527	0	0.53545	0.018805	0	12.62514	0.874973	1.724133
	Std	0	0.597359	0	0	0	0.006928	0	0.090116	0.004936	0	2.99544	0.251541	0.136367
	Median	0	1.599383	0	0	0	0.008985	0	0.416101	0	0	7.303819	0.122234	1.446261
	Rank	1	7	1	1	1	3	1	5	2	1	8	4	6
F12	Mean	1.57E−32	3.266433	2.58E−09	1.316298	2.03E−10	5.786999	0.020076	0.913727	0.039839	0.071258	0.209827	1.499557	0.27462
	Best	1.57E−32	0.952182	4.03E−10	0.768409	5.18E−11	1.035821	0.001225	0.000998	0.01255	0.024086	4.74E−19	0.000107	0.06078
	Worst	1.57E−32	7.381298	7.82E−09	1.644259	3.81E−10	14.12186	0.136764	3.844197	0.086697	0.135	0.930839	5.214001	0.650191
	Std	3.09E−48	2.013998	1.82E−09	0.334527	1.06E−10	4.271963	0.044038	1.317485	0.023485	0.023064	0.338436	1.415344	0.152637
	Median	1.57E−32	2.889094	2.39E−09	1.388009	2.05E−10	4.300599	0.005778	0.419859	0.037873	0.068621	0.080118	1.283982	0.264159
	Rank	1	12	3	10	2	13	4	9	5	6	7	11	8
F13	Mean	1.35E−32	3596.082	1.00E−08	3.12E−31	0.002496	2.714174	0.21439	0.032742	0.513307	1.100895	0.056604	3.604014	2.705127
	Best	1.35E−32	13.78381	1.15E−09	6.52E−32	9.94E−10	2.010439	0.037166	0.006436	4.68E−05	0.587903	4.65E−18	0.009563	1.290667
	Worst	1.35E−32	62,099.16	3.80E−08	5.43E−31	0.025288	3.710223	0.699644	0.091535	0.94917	1.539663	0.957417	12.57304	3.93629
	Std	3.09E−48	15,251.83	9.66E−09	2.48E−31	0.006984	0.613784	0.202038	0.027288	0.283844	0.254715	0.235205	3.336838	0.830601
	Median	1.35E−32	44.18622	6.52E−09	4.00E−31	2.82E−09	2.532635	0.165632	0.02361	0.516634	1.113503	1.78E−17	3.302492	2.864354
	Rank	1	13	3	2	4	11	7	5	8	9	6	12	10
Sum rank		6	53	11	27	15	52	18	39	32	32	44	50	44
Mean rank		1	8.833333	1.833333	4.5	2.5	8.666667	3	6.5	5.333333	5.333333	7.333333	8.333333	7.333333
Total ranking		1	11	2	5	3	10	4	7	6	6	8	9	8

### Evaluation of fixed-dimensional multimodal benchmark functions

The authors evaluated the performance of the proposed MOA and other metaheuristic algorithms on ten fixed-dimension multimodal functions (F14 to F23). The goal was to investigate the algorithms’ ability to balance exploration and exploitation during the search process. The optimization results obtained using MOA and the competitor algorithms are reported in Table 4. Based on the simulation results, MOA is the best optimizer for F14, F15, F21, F22, and F23 functions. For functions F16 to F20, MOA has a similar mean performance compared to some competing algorithms. However, MOA has more favorable values for the std index, indicating a more effective performance in solving these functions. Overall, the analysis of the simulation results indicates that MOA, with its high ability to balance exploration and exploitation, performs better in solving fixed-dimension multimodal functions compared to the competitor algorithms.

**Table 4:** Evaluation results of fixed-dimensional multimodal functions.

$F$		MOA	WSO	AVOA	RSA	MPA	TSA	WOA	MVO	GWO	TLBO	GSA	PSO	GA
F14	Mean	0.998004	1.097319	1.097121	3.105171	1.009791	8.639238	2.568192	0.998016	3.692491	0.998017	3.558763	3.593207	1.048628
	Best	0.998004	0.998004	0.998004	0.998035	0.998004	1.991037	0.998004	0.998004	0.998004	0.998004	0.998004	0.998004	0.998004
	Worst	0.998004	1.991037	2.980121	12.65883	1.233486	15.48955	10.75342	0.998239	10.75342	0.998239	11.85901	12.65883	1.991043
	Std	0	0.336821	0.48842	3.365082	0.058023	5.560947	3.243534	5.80E−05	4.107423	5.79E−05	3.031942	4.170141	0.244469
	Median	0.998004	0.998004	0.998004	2.223887	0.998004	11.70612	0.998004	0.998004	2.980121	0.998004	2.889812	1.991037	0.998004
	Rank	1	7	6	9	4	13	8	2	12	3	10	11	5
F15	Mean	0.000307	0.001357	0.000356	0.001123	0.001207	0.016411	0.000809	0.002645	0.003363	0.000595	0.002351	0.002497	0.015374
	Best	0.000307	0.000307	0.000308	0.000712	0.000309	0.000308	0.000312	0.000308	0.000308	0.000311	0.000886	0.000307	0.000783
	Worst	0.000307	0.020345	0.000732	0.002879	0.001674	0.110173	0.002251	0.020344	0.020345	0.00125	0.006954	0.020345	0.066852
	Std	2.80E−19	0.00493	0.000111	0.000515	0.000603	0.033049	0.000541	0.006677	0.008068	0.000442	0.001506	0.006745	0.017858
	Median	0.000307	0.000309	0.000312	0.001022	0.0016	0.00087	0.000686	0.000681	0.000309	0.000326	0.002169	0.000309	0.01426
	Rank	1	7	2	5	6	13	4	10	11	3	8	9	12
F16	Mean	− 1.03163	− 1.03163	− 1.03163	− 1.02941	− 1.02929	− 1.03005	− 1.03163	− 1.03163	− 1.03163	− 1.03162	− 1.03163	− 1.03163	− 1.03162
	Best	− 1.03163	− 1.03163	− 1.03163	− 1.03161	− 1.03163	− 1.03163	− 1.03163	− 1.03163	− 1.03163	− 1.03163	− 1.03163	− 1.03163	− 1.03163
	Worst	− 1.03163	− 1.0316	− 1.0316	− 1.00003	− 1.00093	− 1.00003	− 1.0316	− 1.0316	− 1.0316	− 1.0316	− 1.0316	− 1.0316	− 1.0316
	Std	2.02E−16	7.63E−06	7.61E−06	0.007703	0.00761	0.007786	7.61E−06	7.61E−06	7.61E−06	7.73E−06	7.61E−06	7.61E−06	8.79E−06
	Median	− 1.03163	− 1.03163	− 1.03163	− 1.03129	− 1.0316	− 1.03163	− 1.03163	− 1.03163	− 1.03163	− 1.03163	− 1.03163	− 1.03163	− 1.03163
	Rank	1	7	3	11	12	10	4	6	5	9	2	3	8
F17	Mean	0.397887	0.397888	0.397888	0.410581	0.398401	0.397925	0.397888	0.397888	0.397889	0.39796	0.397888	0.744291	0.465955
	Best	0.397887	0.397887	0.397887	0.398542	0.397887	0.39789	0.397887	0.397887	0.397887	0.397892	0.397887	0.397887	0.397887
	Worst	0.397887	0.397891	0.397891	0.485175	0.401154	0.398205	0.397892	0.397891	0.397891	0.398172	0.397891	2.788791	1.750826
	Std	0	1.12E−06	1.05E−06	0.021411	0.001054	7.51E−05	1.29E−06	1.05E−06	1.38E−06	7.43E−05	1.05E−06	0.780869	0.333276
	Median	0.397887	0.397887	0.397887	0.403771	0.397974	0.397908	0.397888	0.397888	0.397888	0.397949	0.397887	0.397888	0.397907
	Rank	1	4	2	10	9	7	5	3	6	8	2	12	11
F18	Mean	3	3.003162	3.003163	5.775177	6.161661	11.49645	3.003188	3.003162	3.003175	3.003163	3.003162	3.003162	7.301761
	Best	3	3.000014	3.000014	3.000058	3.013933	3.000021	3.000014	3.000014	3.000018	3.000015	3.000014	3.000014	3.000042
	Worst	3	3.027001	3.027001	31.28671	30.00128	91.94642	3.027003	3.027002	3.027013	3.027004	3.027001	3.027001	34.91828
	Std	1.29E−15	7.01E−03	7.01E−03	9.372117	7.007912	28.84276	7.00E−03	7.01E−03	7.01E−03	7.01E−03	7.01E−03	7.01E−03	11.60624
	Median	3	3.000564	3.000564	3.002285	3.563655	3.001789	3.000572	3.000564	3.000586	3.000564	3.000564	3.000564	3.003009
	Rank	1	2	6	10	11	13	9	5	8	7	4	3	12
F19	Mean	− 3.86278	− 3.86264	− 3.86264	− 3.83682	− 3.72483	− 3.86224	− 3.86029	− 3.86264	− 3.86112	− 3.86154	− 3.86264	− 3.86264	− 3.86248
	Best	− 3.86278	− 3.86278	− 3.86278	− 3.85881	− 3.86278	− 3.86274	− 3.86276	− 3.86278	− 3.86278	− 3.86268	− 3.86278	− 3.86278	− 3.86278
	Worst	− 3.86278	− 3.86221	− 3.86221	− 3.7791	− 3.2931	− 3.85594	− 3.85473	− 3.86221	− 3.85493	− 3.85487	− 3.86221	− 3.86221	− 3.86165
	Std	2.51E−15	1.51E−04	1.51E−04	0.025252	0.151444	0.001642	0.003166	1.51E−04	0.002863	0.002512	1.51E−04	1.51E−04	0.000418
	Median	− 3.86278	− 3.86265	− 3.86265	− 3.84403	− 3.72574	− 3.86259	− 3.86162	− 3.86264	− 3.86258	− 3.86219	− 3.86265	− 3.86265	− 3.86261
	Rank	1	2	3	10	11	6	9	4	8	7	2	2	5
F20	Mean	− 3.322	− 3.30339	− 3.26776	− 2.76501	− 2.53258	− 3.25433	− 3.24918	− 3.2736	− 3.2583	− 3.24203	− 3.32121	− 3.26389	− 3.2276
	Best	− 3.322	− 3.3219	− 3.32153	− 3.06881	− 3.22483	− 3.32105	− 3.32147	− 3.3219	− 3.32189	− 3.31539	− 3.3219	− 3.3219	− 3.32071
	Worst	− 3.322	− 3.20238	− 3.20188	− 1.67045	− 1.78365	− 3.08912	− 3.08873	− 3.20163	− 3.08302	− 3.01276	− 3.32046	− 3.13648	− 2.99698
	Std	4.89E−16	0.047927	0.066814	0.343733	0.37135	0.078284	0.092315	0.066035	0.083885	0.088403	3.71E−04	0.082662	0.085962
	Median	− 3.322	− 3.32122	− 3.3206	− 2.83526	− 2.58954	− 3.26037	− 3.31743	− 3.32109	− 3.3206	− 3.29115	− 3.32126	− 3.32096	− 3.23604
	Rank	1	3	5	12	13	8	9	4	7	10	2	6	11
F21	Mean	− 10.1532	− 8.40566	− 10.1506	− 5.0577	− 7.55876	− 5.92684	− 9.3836	− 8.88417	− 9.38852	− 6.85344	− 7.19449	− 5.62575	− 6.26153
	Best	− 10.1532	− 10.1531	− 10.1532	− 5.06029	− 10.1515	− 10.1294	− 10.1525	− 10.1531	− 10.153	− 9.41091	− 10.1532	− 10.153	− 9.7366
	Worst	− 10.1532	− 2.68523	− 10.1481	− 5.0552	− 5.0552	− 2.61057	− 5.0555	− 5.05519	− 5.05878	− 3.24733	− 2.68523	− 2.6332	− 2.38845
	Std	2.29E−15	3.461007	2.26E−03	2.26E−03	2.261818	3.562346	2.054666	2.479705	2.049661	2.286936	3.807097	3.174503	2.985975
	Median	− 10.1532	− 10.1501	− 10.1509	− 5.05804	− 7.90122	− 5.00071	− 10.1478	− 10.1489	− 10.1495	− 7.31253	− 10.1481	− 5.10141	− 7.0612
	Rank	1	6	2	13	7	11	4	5	3	9	8	12	10
F22	Mean	− 10.4029	− 10.0185	− 10.4006	− 5.09067	− 8.0897	− 6.88561	− 8.1085	− 8.43435	− 10.4001	− 7.94995	− 10.1272	− 6.38464	− 7.37259
	Best	− 10.4029	− 10.4029	− 10.4029	− 5.09298	− 10.4005	− 10.3389	− 10.4025	− 10.4026	− 10.4027	− 10.0595	− 10.4029	− 10.4027	− 9.98289
	Worst	− 10.4029	− 2.75928	− 10.3976	− 5.08767	− 5.08767	− 1.83607	− 1.84121	− 2.77181	− 10.3962	− 4.05144	− 4.93328	− 2.75499	− 2.67923
	Std	3.86E−15	1.882915	2.31E−03	2.31E−03	2.306028	3.864814	3.359301	3.078154	0.002406	1.842456	1.347229	3.8203	2.110814
	Median	− 10.4029	− 10.4004	− 10.4016	− 5.09163	− 9.04577	− 7.4937	− 10.3939	− 10.3982	− 10.4011	− 8.38282	− 10.4008	− 5.11069	− 7.86286
	Rank	1	5	2	13	8	11	7	6	3	9	4	12	10
F23	Mean	− 10.5364	− 10.535	− 10.535	− 5.1325	− 9.15341	− 7.41676	− 8.58402	− 9.46154	− 10.5346	− 8.08721	− 10.2862	− 6.42356	− 6.36296
	Best	− 10.5364	− 10.5363	− 10.5363	− 5.13379	− 10.4492	− 10.4786	− 10.5357	− 10.5363	− 10.5361	− 9.69136	− 10.5363	− 10.5362	− 10.1794
	Worst	− 10.5364	− 10.531	− 10.531	− 5.12847	− 5.12848	− 2.42786	− 1.68387	− 5.13182	− 10.5306	− 4.27265	− 5.55955	− 2.42803	− 2.38964
	Std	3.05E−15	1.62E−03	1.62E−03	1.63E−03	1.62432	3.822669	3.591775	2.42765	0.001644	1.82832	1.226031	4.236135	2.871335
	Median	− 10.5364	− 10.5354	− 10.5354	− 5.13289	− 9.54713	− 10.2895	− 10.5331	− 10.535	− 10.5349	− 8.68008	− 10.5354	− 3.84095	− 6.89094
	Rank	1	2	3	13	7	10	8	6	4	9	5	11	12
Sum rank		10	45	34	106	88	102	67	51	67	74	47	81	96
Mean rank		1	4.5	3.4	10.6	8.8	10.2	6.7	5.1	6.7	7.4	4.7	8.1	9.6
Total ranking		1	3	2	12	9	11	6	5	6	7	4	8	10

**Figure 2:**
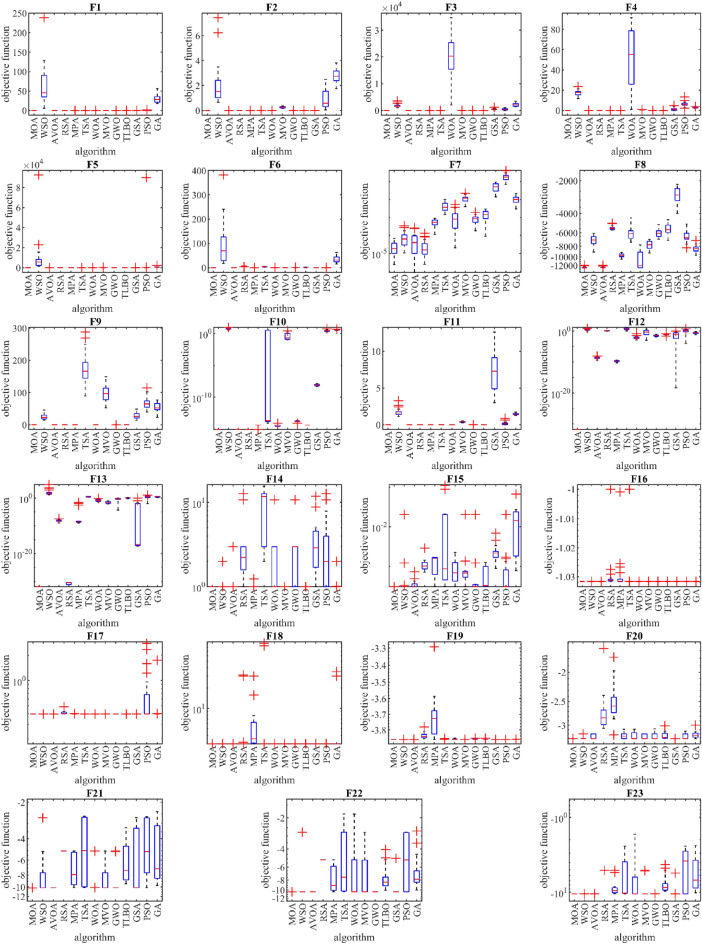
Boxplot of performance of MOA and competitor algorithms in solving F1 to F23.

**Figure 3:**
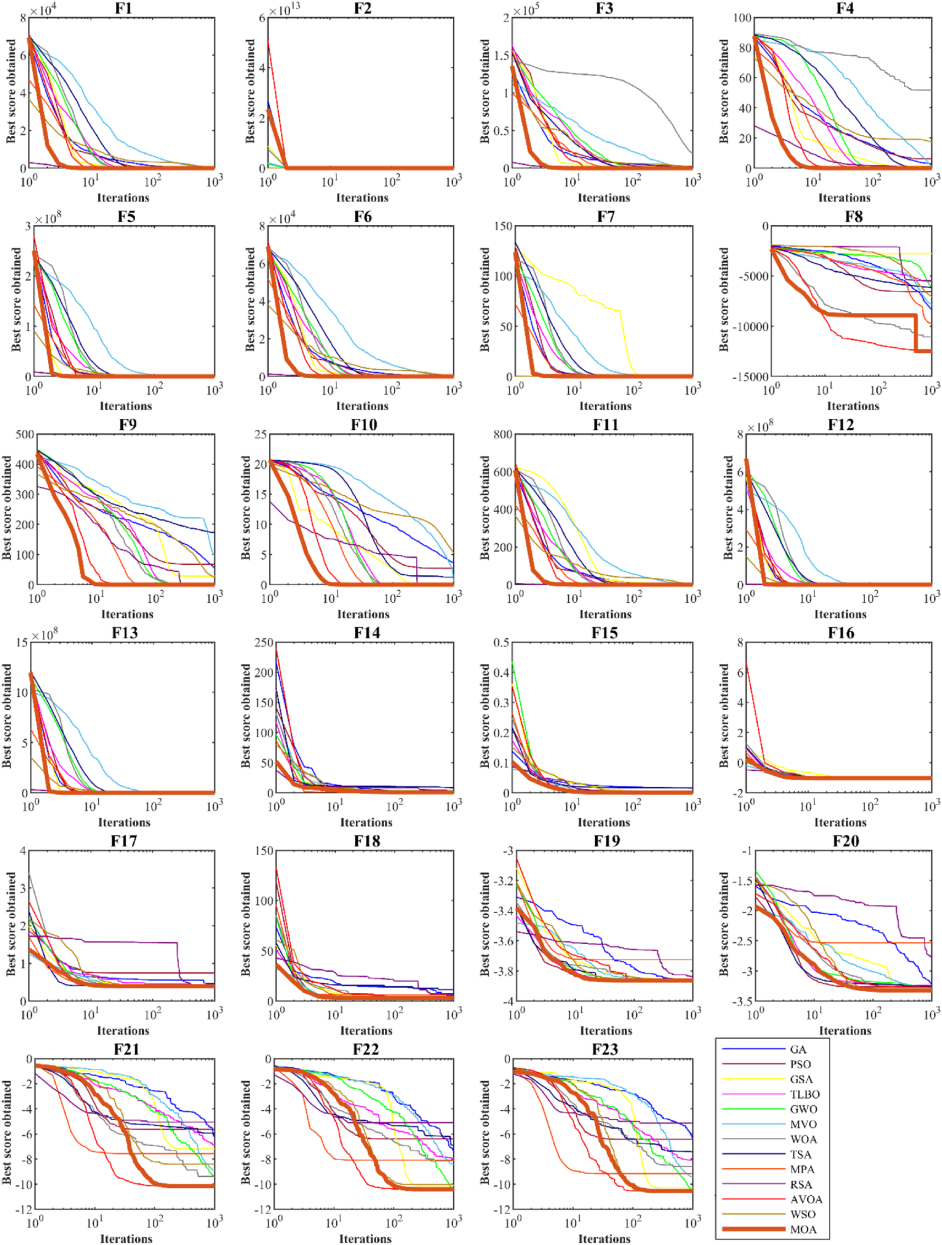
Convergence curves of performance of MOA and competitor algorithms in solving F1 to F23.

Figure 2 shows boxplots of the performance results of MOA and other competing algorithms on functions F1 to F23. The interpretation of the boxplot diagrams is as follows in the functions F1 to F6, F9, and F11. MOA has converged to the global optimum with a standard deviation equal to zero in different executions. This indicates that the proposed algorithm is robust in handling these functions. Also, MOA performed more effectively in dealing with other benchmark functions such as F7, F8, F10, F12, and F23. In addition to providing better values for statistical indicators, it can be seen that the boxplot diagrams of these functions have a smaller area, less dispersion of results in different executions, and a better mean value compared to competitor algorithms. Figure 3 shows the convergence curves of MOA and competitor algorithms in solving functions F1 to F23. The convergence curves show that MOA with a suitable convergence speed, during successive iterations of the algorithm, provided a convenient local search in functions F1 to F7 with the priority of converging to the optimal solution and also without stopping at the local optimum in multimodal functions F8 to F23, the process of optimization and search in the problem-solving space continues.

### CEC 2017 test suite evaluation

**Figure 4:**
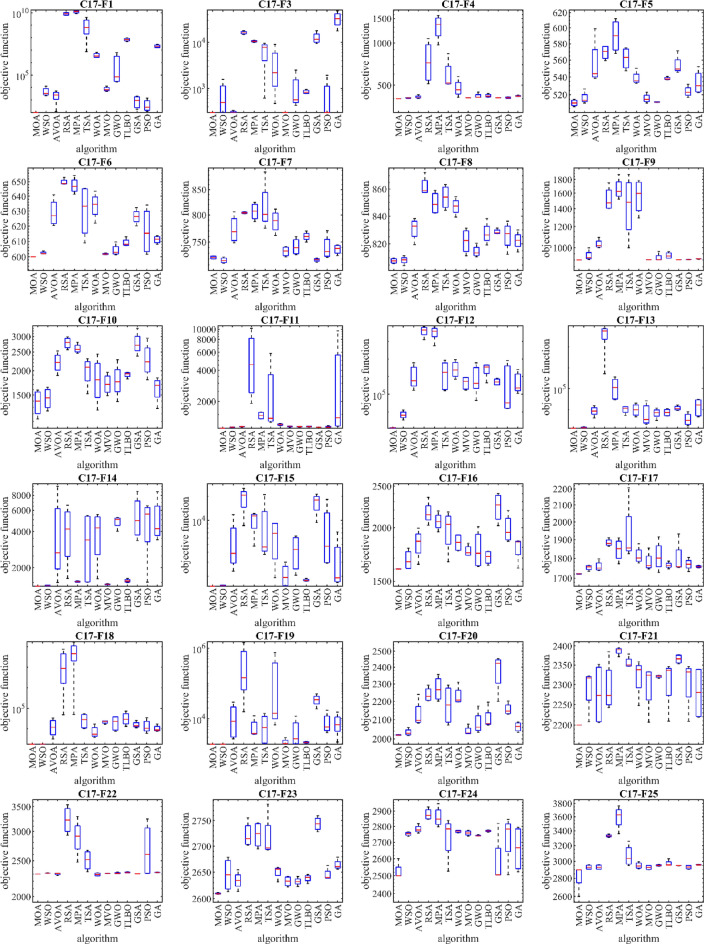

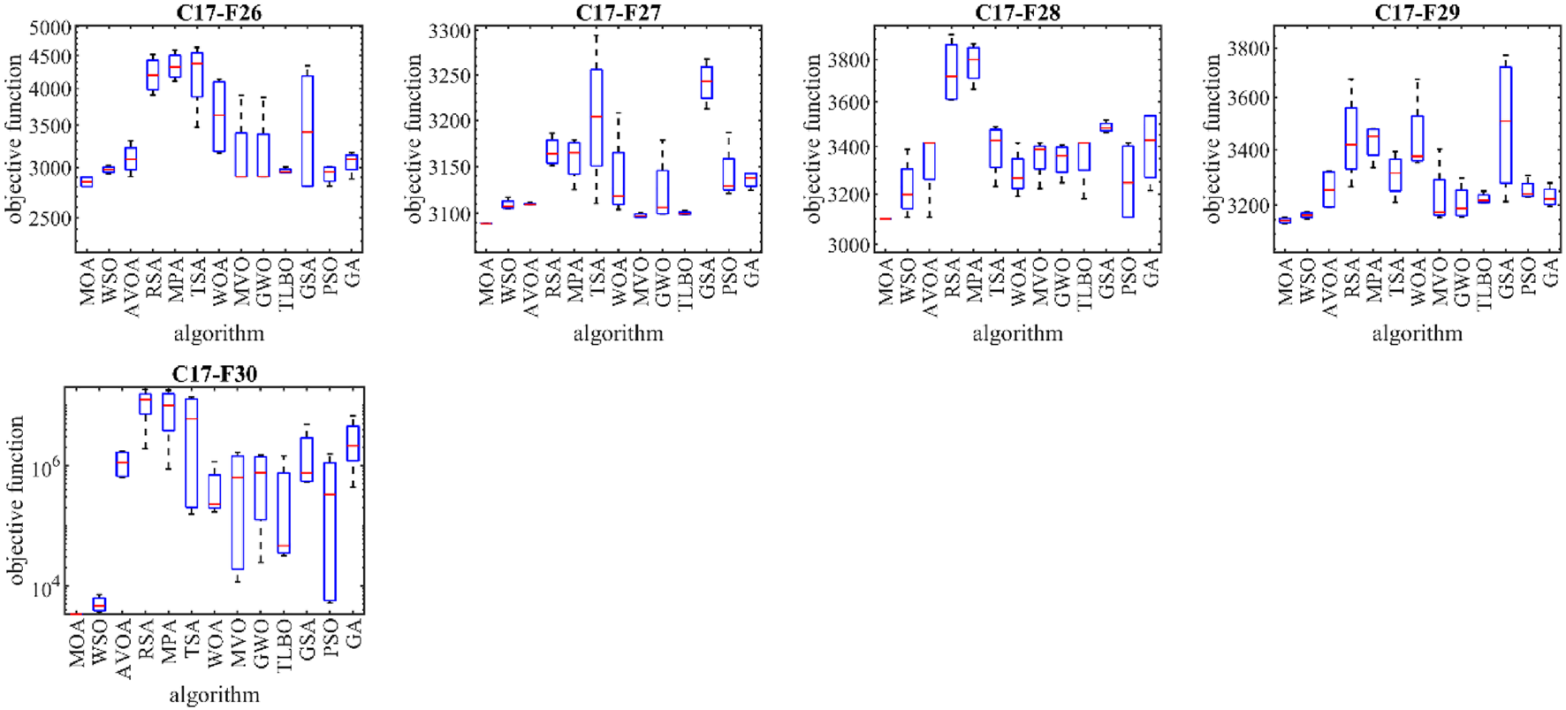
Boxplot of performance of MOA and competitor algorithms in solving CEC 2017 test suite.

**Figure 5:**
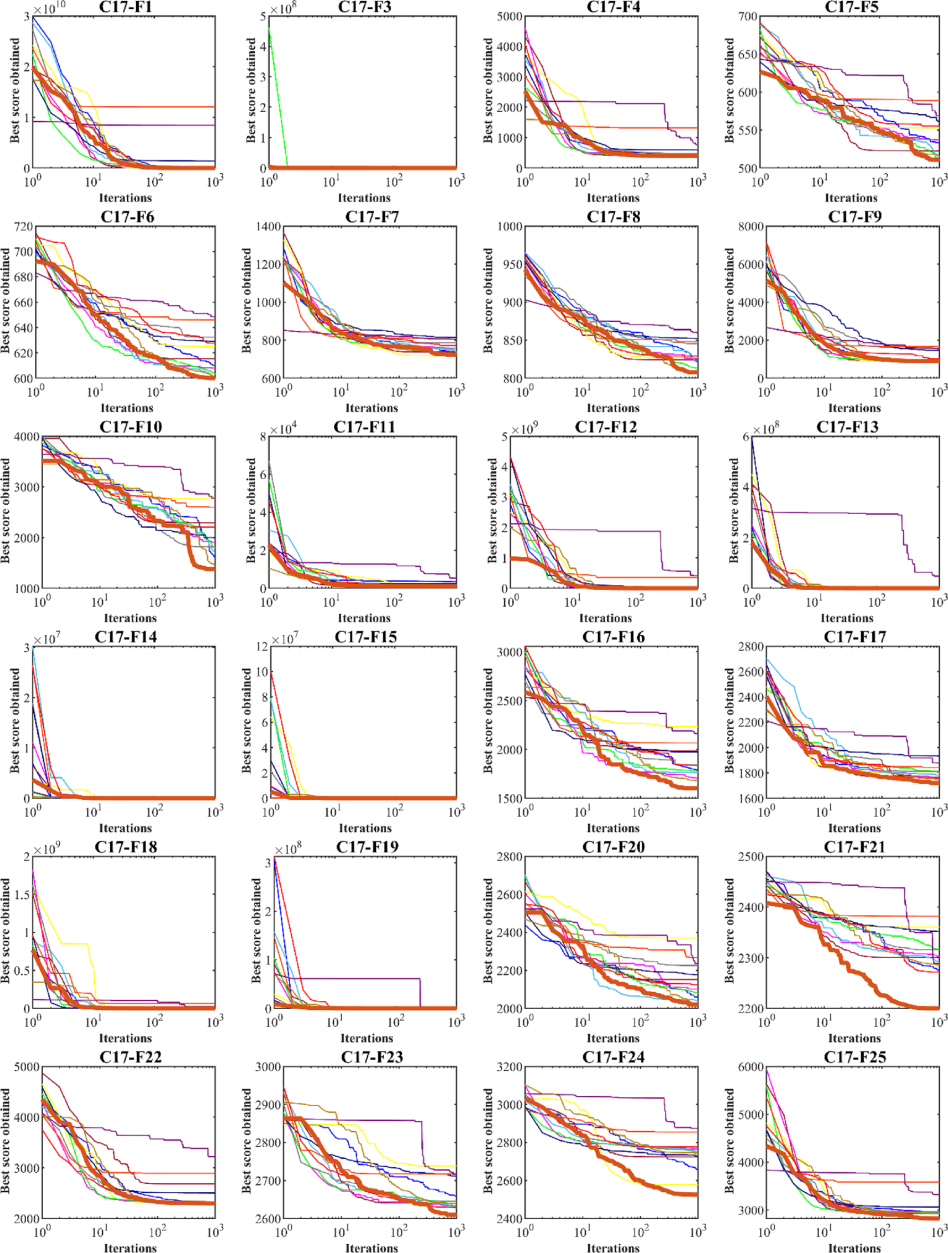

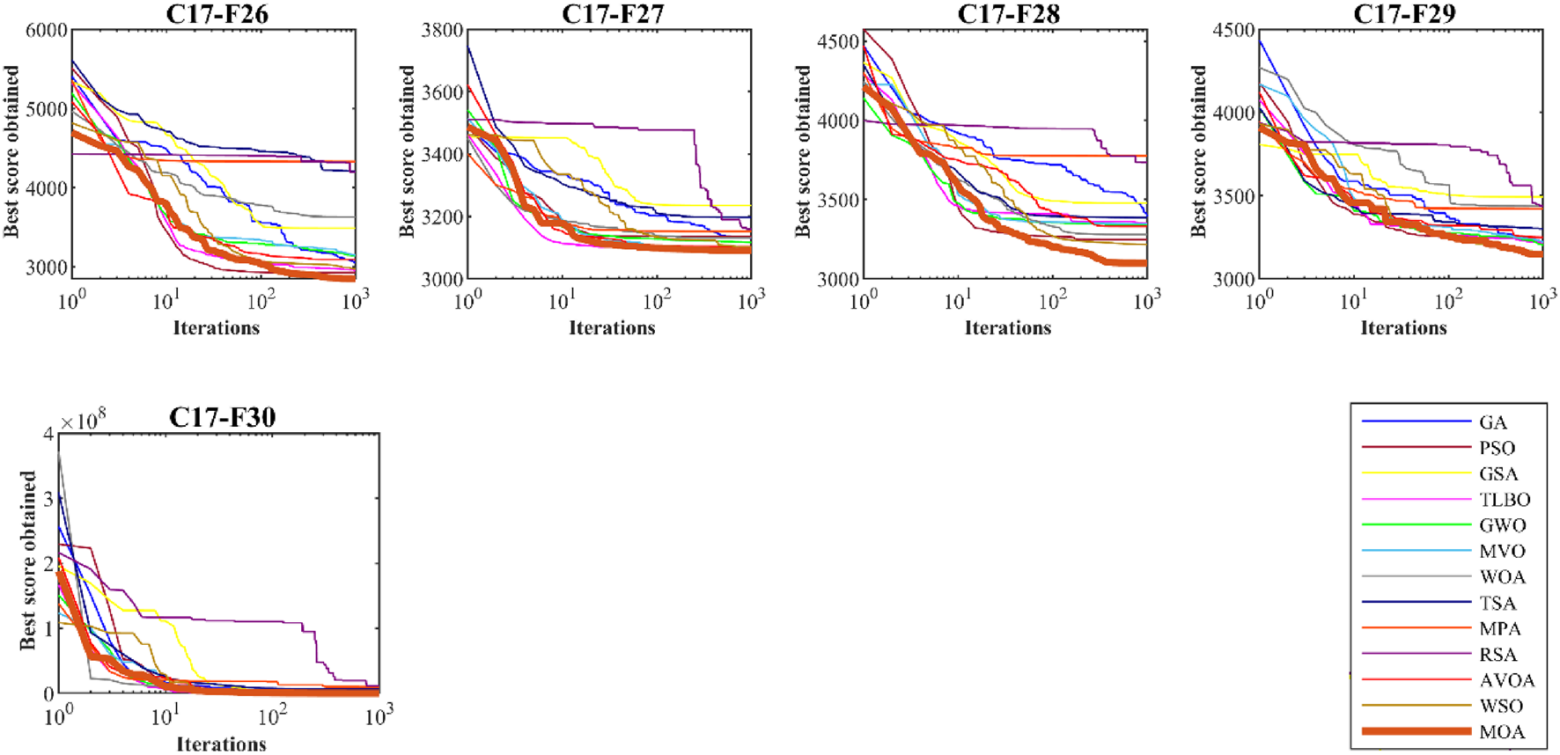
Convergence curves of performance of MOA and competitor algorithms in solving CEC 2017 test suite.

This subsection evaluates MOA’s efficiency in handling the CEC 2017 test suite, which consists of 30 standard benchmark functions (C17–F1 to C17–F30). Results of MOA and competitor algorithms on this suite are reported in Table 5. The boxplot diagrams are shown in Fig. 4 and the convergence curves of metaheuristic algorithms’ performance are drawn in Fig. 5. MOA is the top-performing optimizer for C17–F1, C17–F3 to C17–F6, C17–F8 to C17–F21, and C17–F23 to C17–F30, except for C17–F2 due to its unstable behavior. Overall, the analysis of the optimization results shows that MOA provides better outcomes for most of the benchmark functions and has superior performance compared to competitor algorithms in handling the CEC 2017 test suite. The boxplot diagrams are interpreted in this way, especially in functions C17–F1, C17–F3, C17–F4, C17–F6, C17–F9, C17–F11 to C17–F23, C17–F27, C17–F28, and C17–F30. That MOA with a very low standard deviation and a smaller box area in different implementations has been able to provide more effective and robust performance in handling these functions. The analysis of boxplot diagrams intuitively shows that MOA has provided superior performance compared to competitor algorithms by delivering better results for statistical indicators such as mean and standard deviation. The convergence curves show that in dealing with the unimodal functions C17–F1 and C17–F3, it has converged towards the global optimum with high ability in exploitation and local search at a suitable speed. In dealing with functions C17–F4 to C17–F30, it is evident that MOA moves towards better solutions based on the appropriate ability in exploration during successive iterations, and this process continues until the final iterations.

**Table 5:** Evaluation results of CEC 2017 test suite.

		MOA	WSO	AVOA	RSA	MPA	TSA	WOA	MVO	GWO	TLBBO	GSA	PSO	GA
C17–F1	Mean	100	5394.774	2584.206	8.45E+09	1.21E+10	1.40E+09	3,986,948	7696.359	1,628,024	70,760,095	1009.284	512.6094	20,891,254
	Std	1.70E−05	4824.026	2058.003	2.26E+09	2.78E+09	1.92E+09	1,921,895	3456.125	3,105,598	20,023,090	894.4369	599.4761	6,388,837
	Rank	1	5	4	12	13	11	8	6	7	10	3	2	9
C17–F3	Mean	300	714.2679	309.564	16,206.04	10,517.4	6424.538	3461.475	300.63	1012.924	840.153	12,588.77	704.8032	32,647.85
	Std	8.88E−11	587.2788	13.90441	1266.019	502.0492	3929.786	3742.191	0.026746	982.2947	83.36937	3647.85	798.7056	12,440.17
	Rank	1	5	3	12	10	9	8	2	7	6	11	4	13
C17–F4	Mean	400	404.5583	410.9666	758.4671	1319.753	596.518	477.568	405.7309	418.0512	419.7812	406.4237	406.4037	417.646
	Std	6.61E−08	3.025907	10.68977	261.2167	257.4962	160.2902	69.68358	0.553739	18.8305	11.46374	0.903474	4.885053	4.077261
	Rank	1	2	6	12	13	11	10	3	8	9	5	4	7
C17–F5	Mean	510.9445	516.297	556.3801	569.3022	589.9184	562.0992	538.6935	515.9167	511.8005	538.334	553.9128	523.5634	533.7623
	Std	3.589474	6.77867	28.12457	8.576477	20.89884	13.14489	7.84281	5.005719	0.262822	1.709557	11.49747	6.081188	13.64295
	Rank	1	4	10	12	13	11	8	3	2	7	9	5	6
C17–F6	Mean	600.0006	602.4926	628.775	649.7447	647.3203	630.2	633.6467	601.853	604.1786	609.0722	626.265	616.5693	611.0825
	Std	0.000106	0.860338	9.189882	2.173688	5.364324	17.72122	8.792381	0.482336	3.679604	2.634279	4.898564	15.56608	2.467292
	Rank	1	3	9	13	12	10	11	2	4	5	8	7	6
C17–F7	Mean	722.5537	717.8004	772.8716	804.1864	806.7154	816.0238	787.9268	733.2755	741.7064	759.9908	718.6036	739.647	736.9667
	Std	2.754451	4.781629	25.46749	1.845387	16.60913	47.97126	20.7484	8.666463	14.8834	8.088253	2.978781	21.3286	8.489258
	Rank	3	1	9	11	12	13	10	4	7	8	2	6	5
C17–F8	Mean	807.9597	808.342	830.7956	861.9027	849.5741	853.9328	847.0794	821.9172	814.5836	827.503	828.5749	825.8607	822.2984
	Std	1.794737	2.784854	7.991045	7.146276	8.289771	8.886527	6.094778	8.90872	4.107739	7.875534	1.849425	9.832576	6.346926
	Rank	1	2	9	13	11	12	10	4	3	7	8	6	5
C17–F9	Mean	900	934.7615	1032.844	1524.469	1660.832	1460.543	1567.801	902.0541	918.4551	936.127	901.8	903.1904	908.5115
	Std	3.38E−08	42.78457	41.39303	160.7065	151.4411	373.8084	228.8343	0.283973	32.41159	25.45011	3.39E−10	1.677232	2.09728
	Rank	1	7	9	11	13	10	12	3	6	8	2	4	5
C17–F10	Mean	1379.646	1447.938	2207.952	2783.489	2603.223	2001.444	1822.235	1715.447	1795.333	1910.522	2771.759	2296.61	1622.572
	Std	211.5795	183.5644	262.6804	187.626	144.3814	333.4632	489.0606	207.1481	362.1194	65.28343	379.626	474.1321	237.995
	Rank	1	2	9	13	11	8	6	4	5	7	12	10	3
C17–F11	Mean	1101.505	1126.225	1139.428	5295.611	1465.28	2458.247	1194.559	1142.959	1138.129	1141.094	1124.307	1133.553	3397.024
	Std	1.269139	9.260828	9.105299	3684.331	118.8349	2220.135	27.0935	15.47838	10.55471	11.15396	1.071719	21.2361	4127.228
	Rank	1	3	6	13	10	11	9	8	5	7	2	4	12
C17–F12	Mean	1264.785	7405.993	1,857,668	4.16E+08	3.58E+08	3,075,549	3,660,804	542,782.3	1,736,256	3,029,406	535,746.2	2,052,605	780,029.6
	Std	70.77641	3912.516	2,797,316	2.34E+08	2.40E+08	3,901,116	3,798,891	394,130.1	2,859,725	1,931,222	231,872.9	4,002,080	1,163,873
	Rank	1	2	7	13	12	10	11	4	6	9	3	8	5
C17–F13	Mean	1305.286	1409.721	9255.56	48,319,984	156,456.4	10,757.24	11,271.56	8876.265	7332.801	7770.462	12,151.13	4002.806	17,472.09
	Std	3.253005	90.92971	4795.622	34,341,047	157,875.7	4081.353	7174.125	11,669.59	3592.36	2998.318	3192.702	2832.82	14,149.47
	Rank	1	2	7	13	12	8	9	6	4	5	10	3	11
C17–F14	Mean	1404.229	1421.431	4072.795	4152.507	1530.82	3434.022	3926.646	1454.569	4830.939	1556.091	5458.992	4799.976	5105.294
	Std	3.247945	11.51847	3603.152	2123.556	18.62335	2180.291	1742.542	13.11094	544.8514	52.93744	2301.977	2247.15	2318.283
	Rank	1	2	8	9	4	6	7	3	11	5	13	10	12
C17–F15	Mean	1500.466	1535.347	5460.987	18,776.29	9913.155	8510.701	6231.2	2098.377	4279.24	1792.491	16,658.93	7685.853	3158.503
	Std	0.306769	15.28199	4238.325	6994.636	3414.155	8222.153	3525.437	701.4475	2099.798	61.61513	5065.939	7184.434	2632.158
	Rank	1	2	7	13	11	10	8	4	6	3	12	9	5
C17–F16	Mean	1601.334	1682.445	1839.092	2166.835	2069.018	1978.16	1846.775	1765.09	1780.596	1705.695	2236.048	1987.966	1794.031
	Std	0.862582	90.70794	143.5491	134.8703	95.39254	218.5485	86.6392	53.87892	182.4643	62.21953	173.939	149.8748	121.6675
	Rank	1	2	7	12	11	9	8	4	5	3	13	10	6
C17–F17	Mean	1720.654	1752.533	1758.496	1883.424	1845.692	1935.246	1814.364	1781.303	1811.675	1764.276	1799.808	1769.258	1757.12
	Std	1.727903	13.4588	26.30422	19.61517	58.69379	179.5958	43.64423	48.94268	77.40438	15.90544	87.01531	29.14751	5.458565
	Rank	1	2	4	12	11	13	10	7	9	5	8	6	3
C17–F18	Mean	1800.479	1826.583	15,205.55	23,378,880	64,940,626	30,319.65	7958.805	21,783.12	23,306.93	36,215.28	16,254.63	15,543.93	10,589.09
	Std	0.05863	13.9203	13,293.05	35,070,642	71,191,750	22,370.64	6062.679	3533.925	16,748.96	26,025.44	6315.797	12,751.68	4398.01
	Rank	1	2	5	12	13	10	3	8	9	11	7	6	4
C17–F19	Mean	1900.702	1913.364	12,247.17	436,755.4	5810.262	6837.111	195,324.4	2191.603	4790.23	2138.956	34,704.01	8236.65	7779.356
	Std	0.427842	4.436735	12,323.19	665,048.8	4103.845	5830.044	362,945.7	477.0055	4690.01	116.1022	12,672.72	6067.069	5444.44
	Rank	1	2	10	13	6	7	12	4	5	3	11	9	8
C17–F20	Mean	2019.37	2033.266	2128.562	2238.568	2272.277	2181.009	2230.301	2040.996	2082.269	2106.398	2375.093	2156.253	2062.366
	Std	2.038897	17.8688	73.47092	41.157	70.45386	109.4213	53.19774	23.40831	61.72129	58.81	116.6622	32.31973	24.6195
	Rank	1	2	7	11	12	9	10	3	5	6	13	8	4
C17–F21	Mean	2200	2290.676	2276.057	2293.084	2386.818	2356.454	2320.465	2297.017	2320.752	2307.248	2365.539	2305.033	2279.787
	Std	1.53E−05	54.79291	78.02373	63.24152	10.28605	14.65714	48.88532	59.71873	3.55571	64.48889	11.63849	63.6209	67.40391
	Rank	1	4	2	5	13	11	9	6	10	8	12	7	3
C17–F22	Mean	2300.224	2314.731	2303.994	3227.993	2897.683	2509.825	2294.944	2308.56	2314.187	2322.982	2304.701	2688.169	2322.792
	Std	0.269337	2.09113	17.45317	275.5742	327.6667	156.3429	23.63552	1.362762	11.15749	6.493858	0.197655	452.8454	2.661702
	Rank	2	7	3	13	12	10	1	5	6	9	4	11	8
C17–F23	Mean	2609.635	2645.396	2634.253	2721.782	2722.347	2717.131	2650.59	2632.575	2632.437	2638.587	2743.695	2645.144	2663.978
	Std	1.438651	31.51647	16.5902	24.45716	25.37687	43.37295	12.56156	9.526387	7.560925	7.277304	13.57048	11.51228	9.999389
	Rank	1	7	4	11	12	10	8	3	2	5	13	6	9
C17–F24	Mean	2525.171	2752.267	2782.752	2879.698	2860.669	2733.065	2768.282	2758.003	2741.52	2771.078	2583.9	2729.385	2662.547
	Std	49.73738	12.17657	25.28246	37.66572	65.12911	138.6968	7.684337	16.12647	4.073424	5.870153	155.243	149.9454	139.651
	Rank	1	7	11	13	12	5	9	8	6	10	2	4	3
C17–F25	Mean	2823.318	2929.341	2929.746	3329.029	3593.552	3069.786	2948.464	2926.491	2951.622	2975.276	2948.103	2930.041	2957.947
	Std	147.0641	27.09739	28.65288	18.79409	167.5398	136.9743	37.65274	27.34272	9.693107	37.40484	1.475713	23.64338	4.929348
	Rank	1	3	4	12	13	11	7	2	8	10	6	5	9
C17–F26	Mean	2850.001	2978.809	3100.499	4206.394	4338.354	4215.225	3639.395	3154.31	3147.103	2965.251	3495.814	2931.491	3059.523
	Std	57.04117	36.98457	166.3879	274.9528	212.5982	509.8201	522.609	492.1441	477.1277	29.70273	797.1836	95.12112	124.777
	Rank	1	4	6	11	13	12	10	8	7	3	9	2	5
C17–F27	Mean	3089.072	3109.201	3109.83	3166.048	3158.607	3203.216	3137.305	3097.661	3122.623	3100.337	3241.464	3141.599	3135.664
	Std	0.149314	5.414357	1.041531	15.62356	23.56992	74.88506	47.23808	2.351952	37.48225	1.910065	23.03608	29.93848	8.581193
	Rank	1	4	5	11	10	12	8	2	6	3	13	9	7
C17–F28	Mean	3100	3222.248	3338.191	3742.684	3784.47	3392.824	3284.774	3352.963	3343.541	3356.946	3485.345	3253.957	3402.522
	Std	5.84E−05	118.6261	152.8046	153.8028	95.02999	114.2564	93.65222	87.87781	70.86424	116.2291	23.7622	168.9478	159.4454
	Rank	1	2	5	12	13	9	4	7	6	8	11	3	10
C17–F29	Mean	3146.525	3164.963	3255.733	3444.153	3428.231	3307.865	3443.656	3227.828	3208.231	3222.719	3499.493	3253.765	3229.476
	Std	9.568595	10.20158	70.90286	168.1615	66.62771	76.32693	150.8572	115.446	62.07687	18.88763	261.2242	35.07659	35.98322
	Rank	1	2	8	12	10	9	11	5	3	4	13	7	6
C17–F30	Mean	3400.543	5051.404	1,157,840	11,308,633	9,698,406	6,490,475	446,360.4	731,716.7	758,536.2	393,431.9	1,719,866	557,081.9	2,860,703
	Std	8.742004	1539.75	574,969.4	6,783,947	7,381,265	7,219,521	470,854.1	830,971.6	730,272.4	695,987.5	2,062,222	730,458.6	2,660,945
	Rank	1	2	8	13	12	11	4	6	7	3	9	5	10
Sum rank		32	94	192	343	330	288	241	134	175	187	244	180	199
Mean rank		1.103448	3.241379	6.62069	11.82759	11.37931	9.931034	8.310345	4.62069	6.034483	6.448276	8.413793	6.206897	6.862069
Total rank		1	2	7	13	12	11	9	3	4	6	10	5	8

### Statistical analysis

This subsection presents a statistical analysis comparing the performance of MOA with competitor algorithms to determine the significance of MOA’s superiority. The Wilcoxon signed-rank test^90^, a non-parametric statistical analysis used to detect significant differences between the means of two data samples, is employed to achieve this. The test uses a “$p$-value” index to determine whether there is a significant difference between the two data samples or not.

Table 6 presents the results of the Wilcoxon signed-rank test conducted on the performance of MOA and its competitor algorithms. The test is used to determine if there is a significant difference between the means of two data samples. A $p$-value less than 0.05 indicates that MOA has statistically significant superiority over the corresponding algorithm.

**Table 6:** Wilcoxon signed-rank test results.

Compared algorithms	Unimodal	High-multimodal	Fixed-multimodal	CEC 2017 test suite
MOA vs. WSO	1.85E−24	1.97E−21	3.06E−34	2.04E−18
MOA vs. AVOA	3.02E−11	4.99E−05	2.09E−34	3.69E−21
MOA vs. RSA	4.25E−07	1.63E−11	1.44E−34	1.97E−21
MOA vs. MPA	3.01E−24	1.04E−14	2.09E−34	1.97E−21
MOA vs. TSA	3.01E−24	1.31E−20	1.44E−34	1.97E−21
MOA vs. WOA	2.44E−24	6.13E−11	1.44E−34	3.98E−21
MOA vs. MVO	2.02E−24	1.97E−21	1.44E−34	2.18E−21
MOA vs. GWO	2.02E−24	5.34E−16	1.44E−34	2.54E−21
MOA vs. TLBO	2.02E−24	6.98E−15	1.44E−34	1.97E−21
MOA vs. GSA	2.02E−24	1.97E−21	2.09E−34	5.41E−20
MOA vs. PSO	2.02E−24	1.97E−21	2.09E−34	3.76E−20
MOA vs. GA	2.02E−24	1.97E−21	1.44E−34	1.97E−21

## Discussion

This section discusses the proposed MOA approach’s results, performance, advantages, disadvantages, and other aspects. The MOA algorithm is a population-based metaheuristic algorithm that can provide suitable solutions for optimization problems based on random searches in the problem-solving space. This random search process must be managed at both local and global levels in a way so that by balancing them during the search process, the algorithm can: first, based on the global search, thoroughly scans the problem-solving space in all regions to avoid getting stuck in local optima, Second, based on local search, with careful scanning around promising solutions, converge towards better solutions.

Unimodal functions F1 to F7, as well as C17–F1 and C17–F3 from CEC 2017 test suite, because they do not have local optima, are suitable options to evaluate the ability of local search and exploitation of metaheuristic algorithms. These types of functions have only one extremum, and the primary goal of their optimization is to challenge the ability of metaheuristic algorithms to converge to the global optimum. The optimization results of these functions show that MOA with high exploitation ability has converged to the global optimum in functions F1 to F6, and MOA has converged to solutions very close to the global optimum in handling functions F7, C17–F1, and C17–F3. The high-dimensional multimodal functions F8 to F13 have many local extrema besides the original optimum. For this reason, these functions are suitable options for measuring the ability of metaheuristic algorithms in global search and exploration. The optimization results show that MOA can identify the main optimal area of these functions, especially in handling F9 and F11 functions, which is clearly evident by presenting the global optimum. Fixed-dimension multimodal functions F14 to F23 and functions C17–F4 to C17–F30 from the CEC 2017 test suite challenge the ability of metaheuristic algorithms to balance exploration and exploitation. The optimization results of these functions show that MOA, with a high ability to balance exploration and exploitation, has achieved suitable solutions for these benchmark functions. The analysis of the simulation results indicates the high ability of MOA in exploration, exploitation, and balancing during the search process. The significant statistical superiority of MOA's performance compared to competing algorithms in handling benchmark functions has been confirmed by the Wilcoxon signed-rank test statistical analysis.

The proposed MOA approach has several advantages for global optimization problems. The first advantage of MOA is that there is no control parameter in the design of this algorithm, and therefore there is no need to control the parameters in any way. The second advantage of MOA is its high effectiveness in dealing with various optimization problems in various sciences and complex high-dimensional problems. The third advantage of the MOA is its excellent ability to balance exploration and exploitation in the search process, which allows MOA high-speed convergence to provide suitable values for decision variables in optimization tasks, especially in complex problems. The fourth advantage of the MOA is its powerful performance in handling real-world optimization applications. Against these advantages, the proposed MOA approach also has limitations. The first limitation of MOA, similar to all metaheuristic algorithms, is that there is no guarantee of achieving the global optimum using it due to the random search nature. The second limitation of MOA is that, based on the NFL theorem, there is always a possibility that newer metaheuristic algorithms will be designed to perform better than MOA. The third limitation of MOA is that it cannot be claimed that MOA is the best optimizer for all optimization tasks.

### MOA for real-world applications

This section evaluates the performance of MOA in solving real-world optimization problems. Specifically, the proposed MOA approach is implemented on four engineering design optimization problems: tension/compression spring (TCS) design, welded beam (WB) design, speed reducer (SR) design, and pressure vessel (PV) design. The mathematical model and full description of these real-world applications are provided for TCS and WB in Ref.^91^, for SR in Ref.^92, 93^, and for PV in Ref.^94^.

**Figure 6:**
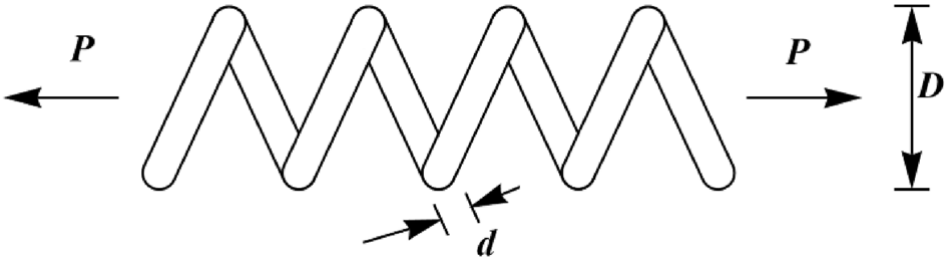
Schematic of the TCS design.

The TCS problem is a design challenge in real-world applications to minimize the weight of the tension/compression spring. The schematic of this design is shown in Fig. 6. Its mathematical model is as follows:

$$Consider: X=\left[{x}_{1}, {x}_{2}, {x}_{3} \right]=\left[d, D, P\right],$$

$$Minimize: f\left(x\right)=\left({x}_{3}+2\right){x}_{2}{x}_{1}^{2}.$$

Subject to:

$${g}_{1}\left(x\right)= 1-\frac{{x}_{2}^{3}{x}_{3}}{71,785{x}_{1}^{4}} \le 0, \; {g}_{2}\left(x\right)=\frac{4{x}_{2}^{2}-{x}_{1}{x}_{2}}{12,566({x}_{2}{x}_{1}^{3})}+\frac{1}{5108{x}_{1}^{2}}-1\le 0,$$

$${g}_{3}\left(x\right)= 1-\frac{140.45{x}_{1}}{{x}_{2}^{2}{x}_{3}}\le 0, \; {g}_{4}\left(x\right)=\frac{{x}_{1}+{x}_{2}}{1.5}-1 \le 0.$$

With

$$0.05\le {x}_{1}\le 2, {0.25\le x}_{2}\le 1.3\text{ and }2\le {x}_{3}\le 15.$$

**Figure 7:**
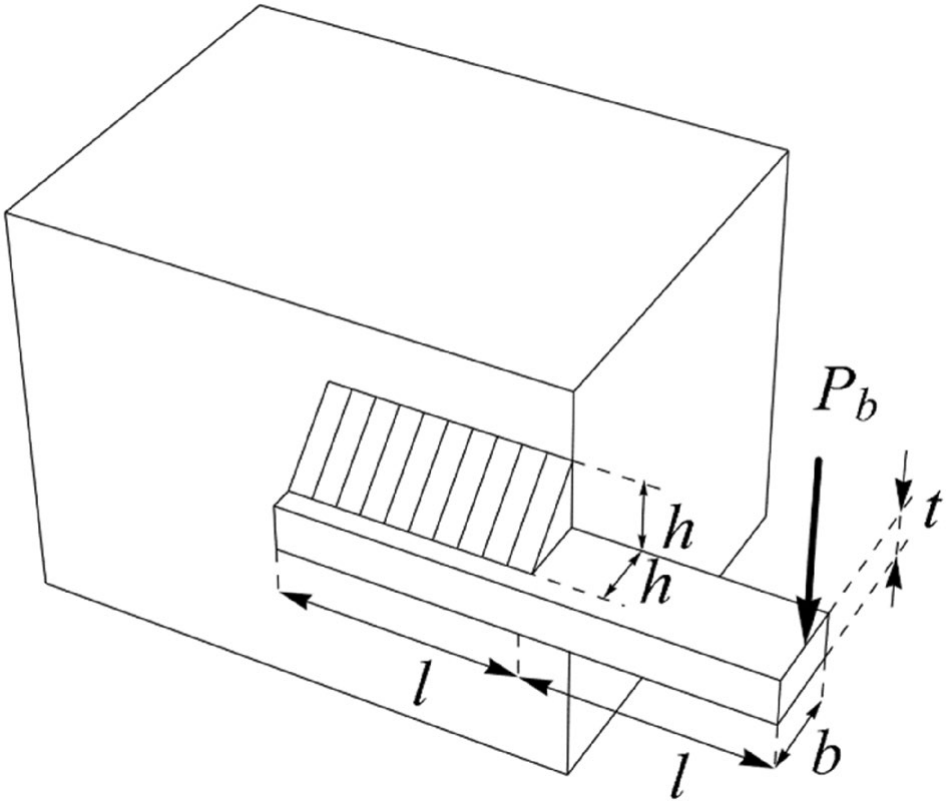
Schematic of the WB design.

The WB problem is a real-world application in engineering to minimize the welded beam’s fabrication cost. The schematic of this design is shown in Fig. 7. Its mathematical model is as follows:

$$Consider: \,X=\left[{x}_{1}, {x}_{2}, {x}_{3}, {x}_{4}\right]=\left[h, l, t, b\right].$$

$$Minimize: \,f\left(x\right)=1.10471{x}_{1}^{2}{x}_{2}+0.04811{x}_{3}{x}_{4} \left(14.0+{x}_{2}\right).$$

Subject to:

$${g}_{1}\left(x\right)= \tau \left(x\right)-13,600 \le 0, \; {g}_{2}\left(x\right)= \sigma \left(x\right)-30,000 \le 0,$$

$${g}_{3}\left(x\right)= {x}_{1}-{x}_{4}\le 0, \; {g}_{4}(x) = 0.10471{x}_{1}^{2}+0.04811{x}_{3}{x}_{4} (14+{x}_{2})-5.0 \le 0,$$

$${g}_{5}\left(x\right)= 0.125 - {x}_{1}\le 0, \; {g}_{6}\left(x\right)= \delta \left(x\right)- 0.25 \le 0,$$

$${g}_{7}\left(x\right)= 6000 - {p}_{c} \left(x\right)\le 0,$$

where

$$\tau \left(x\right)=\sqrt{{\left({\tau }^{^{\prime}}\right)}^{2}+\left(2\tau {\tau }^{^{\prime}}\right)\frac{{x}_{2}}{2R}+{\left(\tau "\right)}^{2} }, {\tau }^{^{\prime}}=\frac{6000}{\sqrt{2}{x}_{1}{x}_{2}}, \tau "=\frac{MR}{J},$$

$$M=6000\left(14+\frac{{x}_{2}}{2}\right), R=\sqrt{\frac{{x}_{2}^{2}}{4}+{\left(\frac{{x}_{1}+{x}_{3}}{2}\right)}^{2}},$$

$$J=2\sqrt{2}{x}_{1}{x}_{2}\left[\frac{{x}_{2}^{2}}{12}+{\left(\frac{{x}_{1}+{x}_{3}}{2}\right)}^{2}\right] , \sigma \left(x\right)=\frac{504000}{{x}_{4}{x}_{3}^{2}},$$

$$\delta \left(x\right)=\frac{65856000}{\left(30\times 1{0}^{6}\right){x}_{4}{x}_{3}^{3}} , {p}_{c} \left(x\right)=\frac{4.013\left(30\times 1{0}^{6}\right){x}_{3}{x}_{4}^{3}}{6\times 196}\left(1-\frac{{x}_{3}}{28}\sqrt{\frac{30\times 1{0}^{6}}{4(12\times 1{0}^{6})}}\right) .$$

With

$$0.1\le {x}_{1}, {x}_{4}\le 2 \text{ and }0.1\le {x}_{2}, {x}_{3}\le 10.$$

**Figure 8:**
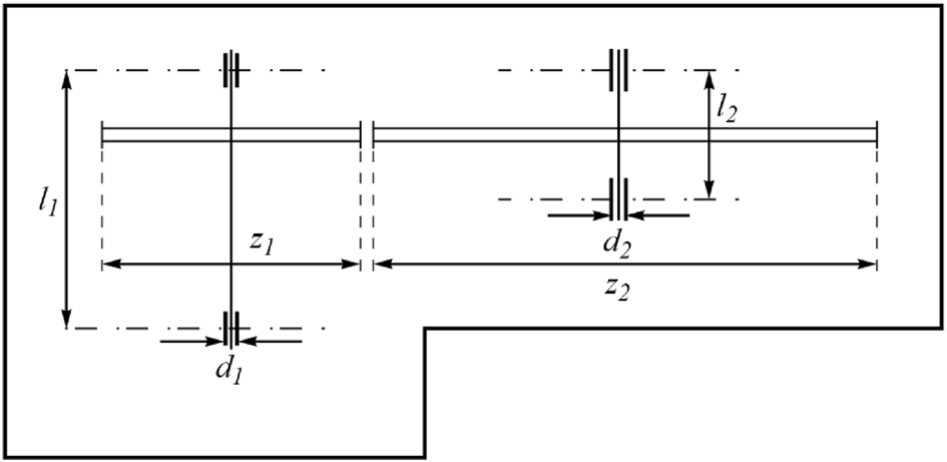
Schematic of the SR design.

The SR problem is an engineering subject whose design goal is to minimize the weight of the speed reducer. The schematic of this design is shown in Fig. 8. Its mathematical model is as follows:

$$Consider: X=\left[{x}_{1,} {x}_{2}, {x}_{3}, {x}_{4}, {x}_{5}{ ,x}_{6} ,{x}_{7}\right]=\left[b, m, p, {l}_{1}, {l}_{2}, {d}_{1}, {d}_{2}\right].$$

$$Minimize: f\left(x\right)=0.7854{x}_{1}{x}_{2}^{2}\left(3.3333{x}_{3}^{2}+14.9334{x}_{3}-43.0934\right)-1.508{x}_{1}\left({x}_{6}^{2}+{x}_{7}^{2}\right)+7.4777\left({x}_{6}^{3}+{x}_{7}^{3}\right)+0.7854\left({x}_{4}{x}_{6}^{2}+{x}_{5}{x}_{7}^{2}\right).$$

Subject to:

$${g}_{1}\left(x\right)=\frac{27}{{x}_{1}{x}_{2}^{2}{x}_{3}}-1 \le 0, \; {g}_{2}\left(x\right)=\frac{397.5}{{x}_{1}{x}_{2}^{2}{x}_{3}}-1\le 0,$$

$${g}_{3}\left(x\right)=\frac{1.93{x}_{4}^{3}}{{x}_{2}{x}_{3}{x}_{6}^{4}}-1\le 0, \; {g}_{4}\left(x\right)=\frac{1.93{x}_{5}^{3}}{{x}_{2}{x}_{3}{x}_{7}^{4}}-1 \le 0,$$

$${g}_{5}\left(x\right)=\frac{1}{110{x}_{6}^{3}}\sqrt{{\left(\frac{745{x}_{4}}{{x}_{2}{x}_{3}}\right)}^{2}+16.9\times {10}^{6}}-1\le 0,$$

$${g}_{6}(x) = \frac{1}{85{x}_{7}^{3}}\sqrt{{\left(\frac{745{x}_{5}}{{x}_{2}{x}_{3}}\right)}^{2}+157.5\times {10}^{6}}-1 \le 0,$$

$${g}_{7}\left(x\right)=\frac{{x}_{2}{x}_{3}}{40}-1 \le 0, \; {g}_{8}\left(x\right)=\frac{{5x}_{2}}{{x}_{1}}-1 \le 0,$$

$${g}_{9}\left(x\right)=\frac{{x}_{1}}{12{x}_{2}}-1 \le 0, \; {g}_{10}\left(x\right)=\frac{{1.5x}_{6}+1.9}{{x}_{4}}-1 \le 0,$$

$${g}_{11}\left(x\right)=\frac{{1.1x}_{7}+1.9}{{x}_{5}}-1 \le 0.$$

With

$$2.6\le {x}_{1}\le 3.6, 0.7\le {x}_{2}\le 0.8, 17\le {x}_{3}\le 28, 7.3\le {x}_{4}\le 8.3, 7.8\le {x}_{5}\le 8.3, 2.9\le {x}_{6}\le 3.9, \text{ and } 5\le {x}_{7}\le 5.5 .$$

**Figure 9:**
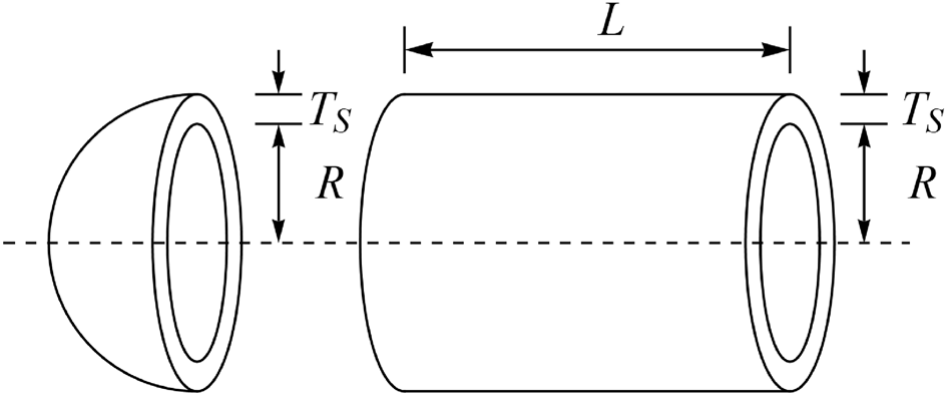
Schematic of the PV design.

The PV problem is a real-world application to minimize the total cost of the design. This design is shown in Fig. 9. Its mathematical model is as follows:

$$Consider: X=\left[{x}_{1}, {x}_{2}, {x}_{3}, {x}_{4}\right]=\left[{T}_{s}, {T}_{h}, R, L\right],$$

$$Minimize: f\left(x\right)=0.6224{x}_{1}{x}_{3}{x}_{4}+1.778{x}_{2}{x}_{3}^{2}+3.1661{x}_{1}^{2}{x}_{4}+19.84{x}_{1}^{2}{x}_{3}.$$

Subject to:

$${g}_{1}\left(x\right)= -{x}_{1}+0.0193{x}_{3} \le 0, \; {g}_{2}\left(x\right)=-{x}_{2}+0.00954{x}_{3}\le 0,$$

$${g}_{3}\left(x\right)=-\pi {x}_{3}^{2}{x}_{4}-\frac{4}{3}\pi {x}_{3}^{3}+1,296,000\le 0, \; {g}_{4}\left(x\right)={x}_{4}-240 \le 0.$$

With

$$0\le {x}_{1},{x}_{2}\le 100 {\text{ and } 10\le x}_{3},{x}_{4}\le 200.$$

**Figure 10:**
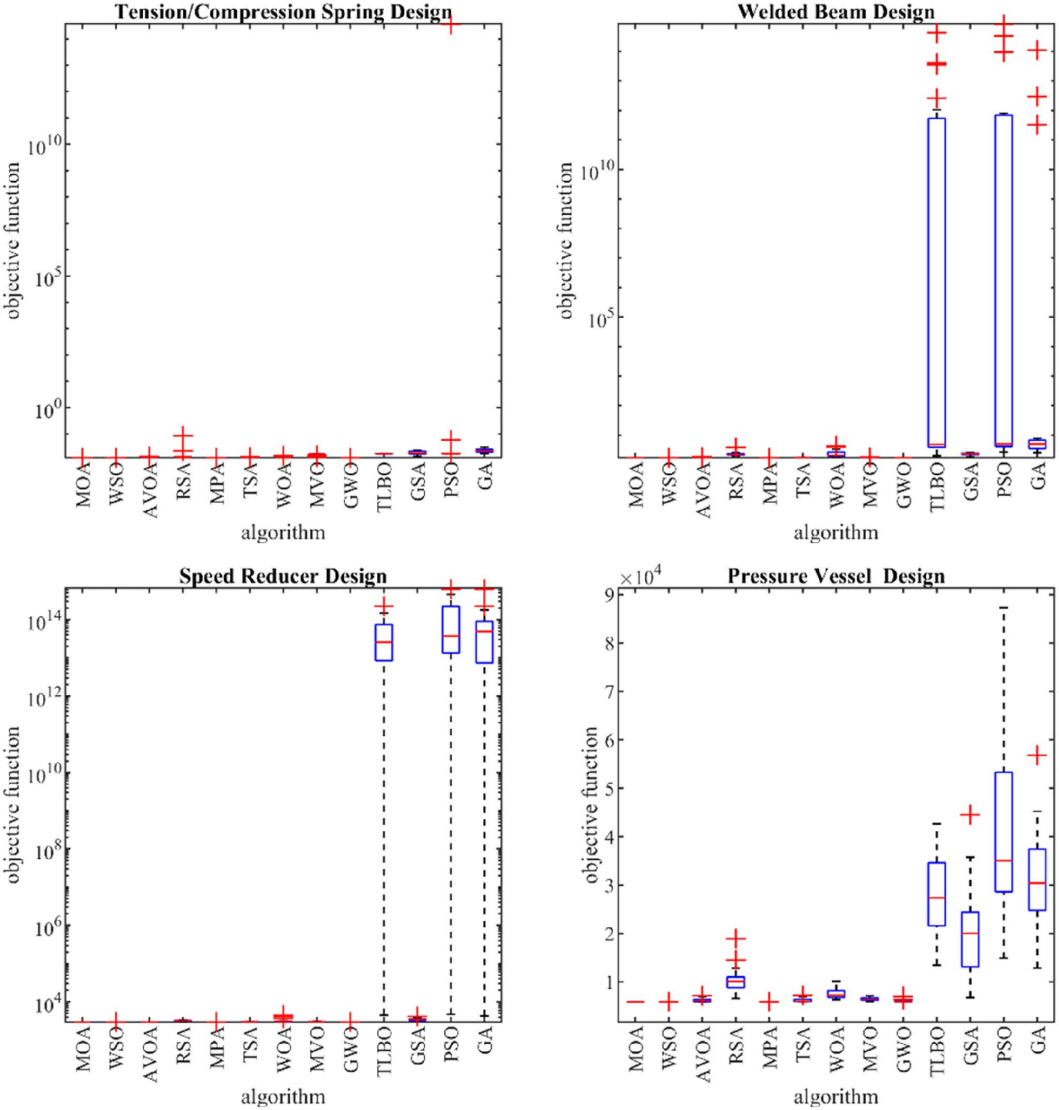
Boxplots of MOA and competitor algorithms performances on the real-world application.

Table 7 presents the optimization results for four engineering design problems, namely tension/compression spring (TCS), welded beam (WB), speed reducer (SR), and pressure vessel (PV), using MOA and competitor algorithms. Figure 10 shows the boxplot diagrams resulting from the performance of MOA and competitor algorithms in solving these four problems. The simulation results show that MOA achieved the best objective function values for all four issues: $2996.348$ for TCS, $5882.901$ for WB, $1.724852$ for SR, and $0.012665$ for PV. The statistical indicators also support MOA’s superiority over competing algorithms. Thus, it can be concluded that the proposed MOA approach is an effective optimizer for real-world optimization problems.

**Table 7:** Evaluation results of real-world applications.

DP		MOA	WSO	AVOA	RSA	MPA	TSA	WOA	MVO	GWO	TLBO	GSA	PSO	GA
TCS	Mean	2996.348	2996.35	3001.338	3228.269	2996.348	3029.188	3248.642	3031.532	3004.404	4.90E+13	3505.394	1.30E+14	8.83E+13
	Best	2996.348	2996.348	2996.351	3093.198	2996.348	3012.411	3008.371	3004.927	2999.069	4504.467	3266.506	4752.969	4315.817
	Worst	2996.348	2996.367	3008.343	3327.121	2996.348	3045.978	4500.267	3063.778	3011.167	2.24E+14	4125.528	6.32E+14	6.25E+14
	Std	9.43E−13	0.004326	3.937294	58.52092	8.00E−06	8.243755	409.5635	16.30123	3.323438	5.95E+13	213.9233	1.81E+14	1.43E+14
	Median	2996.348	2996.349	3001.263	3218.466	2996.348	3028.778	3129.077	3032.788	3004.336	2.54E+13	3469.71	3.70E+13	4.92E+13
	Rank	1	3	4	8	2	6	9	7	5	11	10	13	12
WB	Mean	5882.901	5882.913	6238.696	10,460.35	5882.901	6211.23	7701.624	6456.848	6059.174	28,155.59	20,951.89	41,692.38	31,626.02
	Best	5882.901	5882.901	5882.908	6585.53	5882.901	5908.842	6341.473	5926.502	5889.127	13,439.96	6749.396	14,907.8	12,869.83
	Worst	5882.901	5883.136	7172.714	18,955.69	5882.901	7227.766	10,119.28	7130.798	7047.823	42,670.02	44,562.53	87,257.71	56,777.36
	Std	1.89E−12	0.053087	375.6734	2769.787	2.92E−05	391.0596	1189.411	335.6614	340.3287	8594.949	10,085.92	20,472.23	10,444.33
	Median	5882.901	5882.901	6168.259	10,090.05	5882.901	5980.143	7256.607	6431.211	5905.019	27,378.62	20,033.44	35,015.73	30,429.89
	Rank	1	3	6	9	2	5	8	7	4	11	10	13	12
SR	Mean	1.724852	1.724852	1.744873	2.259737	1.724852	1.742252	2.38973	1.74478	1.727052	2.51E+13	2.300867	6.71E+13	5.60E+12
	Best	1.724852	1.724852	1.724895	1.916195	1.724852	1.732511	1.791035	1.729229	1.725501	1.974231	1.769807	2.653772	2.554918
	Worst	1.724852	1.724852	1.797824	3.780976	1.724852	1.748606	4.271542	1.775473	1.730851	4.24E+14	2.573375	8.13E+14	1.09E+14
	Std	6.90E−16	2.38E−09	0.022106	0.398892	2.35E−08	0.005107	0.731538	0.013222	0.00159	9.56E+13	0.199567	1.95E+14	2.45E+13
	Median	1.724852	1.724852	1.736808	2.176518	1.724852	1.743128	2.030166	1.741371	1.726389	4.765774	2.312585	5.045862	4.938982
	Rank	1	2	7	8	3	5	10	6	4	12	9	13	11
PV	Mean	0.012665	0.012666	0.012983	0.017313	0.012665	0.012908	0.013404	0.01667	0.012716	0.017862	0.019409	3.57E+13	0.023509
	Best	0.012665	0.012665	0.012667	0.01303	0.012665	0.012711	0.012687	0.01289	0.012688	0.017327	0.014155	0.017262	0.017901
	Worst	0.012665	0.012671	0.013992	0.085576	0.012665	0.013275	0.015204	0.017548	0.012735	0.018413	0.024197	3.57E+14	0.031971
	Std	9.85E−19	1.21E−06	0.000377	0.016373	3.06E−09	0.000142	0.000858	0.001416	1.08E−05	0.000328	0.003262	1.11E+14	0.003669
	Median	0.012665	0.012665	0.012837	0.013207	0.012665	0.012915	0.013092	0.01729	0.01272	0.017807	0.019092	0.017262	0.022746
	Rank	1	3	6	9	2	5	7	8	4	10	11	13	12
Sum rank		4	11	23	34	9	21	34	28	17	44	40	52	47
Mean rank		1	2.75	5.75	8.5	2.25	5.25	8.5	7	4.25	11	10	13	11.75
Total ranking		1	3	6	8	2	5	8	7	4	10	9	12	11

## Conclusion and future works

The novelty and innovation of this article are in introducing a new metaheuristic algorithm called Mother Optimization Algorithm (MOA), inspired by the interactions between a mother and her children in three phases: education, advice, and upbringing. First, the implementation of MOA is explained, and its steps are mathematically modeled. Then, the proposed approach is evaluated on 52 benchmark functions, including unimodal, high-dimensional multimodal, fixed-dimensional multimodal, and CEC 2017 test suite. The optimization results of unimodal functions showed that MOA has high exploitation ability and local search in converging towards the global optimum. The optimization results of high-dimensional multimodal functions showed that MOA with high exploration and global search ability could discover the main optimal area in the problem-solving space by avoiding getting stuck in local optima. The optimization results of fixed-dimensional multimodal and CEC 2017 test set demonstrate the high efficiency of MOA in solving optimization problems by maintaining a balance between exploration and exploitation strategies. Furthermore, the performance of MOA is compared to twelve well-known metaheuristic algorithms, and it is shown to outperform most of them in terms of providing more appropriate solutions. Finally, MOA is tested on four engineering design problems, and the results indicate its effectiveness in handling real-world applications. The statistical analysis obtained from the Wilcoxon signed-rank test showed that MOA has a significant statistical superiority in the competition with twelve well-known compared metaheuristic algorithms in handling the optimization problems studied in this paper.

The proposed MOA approach opens up several research possibilities for further studies. One of the most promising research areas is the development of binary and multi-objective versions of the proposed approach. Another potential direction for future work is the application of MOA to optimization problems in various fields and real-world scenarios.

## Data Availability

All data generated or analyzed during this study are included directly in the text of this submitted manuscript. There are no additional external files with datasets.
